# Predictive biomarkers for metachronous gastric cancer development after endoscopic resection of early gastric cancer

**DOI:** 10.1002/cam4.70104

**Published:** 2024-08-22

**Authors:** Bokyung Kim, Harim Chun, Jongwon Lee, Miree Park, Yoonjin Kwak, Jung Mogg Kim, Sang Gyun Kim, Ji Kon Ryu, Jungmin Choi, Soo‐Jeong Cho

**Affiliations:** ^1^ Department of Internal Medicine and Liver Research Institute Seoul National University Hospital, Seoul National University College of Medicine Seoul Korea; ^2^ Department of Biomedical Sciences Korea University College of Medicine Seoul Korea; ^3^ Department of Pathology Seoul National University Hospital Seoul Korea; ^4^ Department of Microbiology Hanyang University College of Medicine Seoul Korea

**Keywords:** biomarkers, endoscopic resection, gastric cancer, RNA sequencing, whole exome sequencing

## Abstract

**Objectives:**

We aimed to identify predictive markers for metachronous gastric cancer (MGC) in early gastric cancer (EGC) patients curatively treated with endoscopic submucosal dissection (ESD).

**Materials and Methods:**

From EGC patients who underwent ESD, bulk RNA sequencing was performed on non‐cancerous gastric mucosa samples at the time of initial EGC diagnosis. This included 23 patients who developed MGC, and 23 control patients without additional gastric neoplasms for over 3 years (1:1 matched by age, sex, and *Helicobacter pylori* infection state). Candidate differentially‐expressed genes were identified, from which biomarkers were selected using real‐time quantitative polymerase chain reaction and cell viability assays using gastric cell lines. An independent validation cohort of 55 MGC patients and 125 controls was used for marker validation. We also examined the severity of gastric intestinal metaplasia, a known premalignant condition, at initial diagnosis.

**Results:**

From the discovery cohort, 86 candidate genes were identified of which *KDF1* and *CDK1* were selected as markers for MGC, which were confirmed in the validation cohort. *CERB5* and *AKT2* isoform were identified as markers related to intestinal metaplasia and were also highly expressed in MGC patients compared to controls (*p* < 0.01). Combining these markers with clinical data (age, sex, *H. pylori* and severity of intestinal metaplasia) yielded an area under the curve (AUC) of 0.91 (95% CI, 0.85‐0.97) for MGC prediction.

**Conclusion:**

Assessing biomarkers in non‐cancerous gastric mucosa may be a useful method for predicting MGC in EGC patients and identifying patients with a higher risk of developing MGC, who can benefit from rigorous surveillance.

## Graphical abstract

Patients with early gastric cancer (EGC) who undergo endoscopic resection are at high risk for developing metachronous gastric cancer (MGC). In this study, we discovered and validated two genes, KDF1 and CDK1, from noncancerous gastric mucosa of EGC patients, showing high predictive accuracy for future MGC development. CREB5 and the AKT2 isoform were identified as indicators of the severity of gastric intestinal metaplasia, a premalignant condition, also demonstrating high predictive accuracy for MGC development.

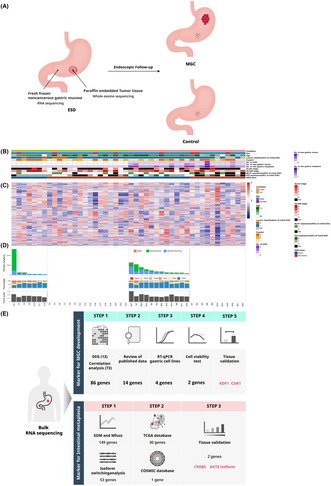

## 1 INTRODUCTION

Gastric cancer is the fifth most common and third most deadly cancer worldwide.^1^ With the early detection of gastric cancer, endoscopic submucosal dissection (ESD) has been widely accepted in the treatment of early gastric cancer (EGC).^2^ However, a major concern for patients undergoing ESD is the development of metachronous gastric cancer (MGC), which refers to the occurrence of new gastric cancer in areas other than the primary site.^3^ Patients with EGC who undergo ESD are known to have a high risk for MGC development, with incidence reported between 2.7% and 15.6% over 2–7 years of follow‐up.^3^, ^4^, ^5^, ^6^ Several previous studies have reported risk factors for MGC development, including old age, male sex, multiple initial cancers, persistent *Helicobacter pylori* (*H. pylori*) infection, family history of gastric cancer, severe gastric mucosal atrophy, and intestinal metaplasia.^6^, ^7^, ^8^, ^9^, ^10^, ^11^ However, little is known regarding genomic risk factors for developing MGC.

Identifying biomarkers capable of predicting MGC development would be of major importance considering the high incidence of MGC and the increasing implementation of ESD. This would enable tailored surveillance strategies specific to the patient's risk profile and offer insights into the mechanisms underlying MGC occurrence and gastric cancer development. In this regard, this study aimed to develop and validate predictive biomarkers for MGC development in patients with EGC who undergo ESD.

## 2 MATERIALS AND METHODS

### 2.1 Study design and patient cohorts

**FIGURE 1:**
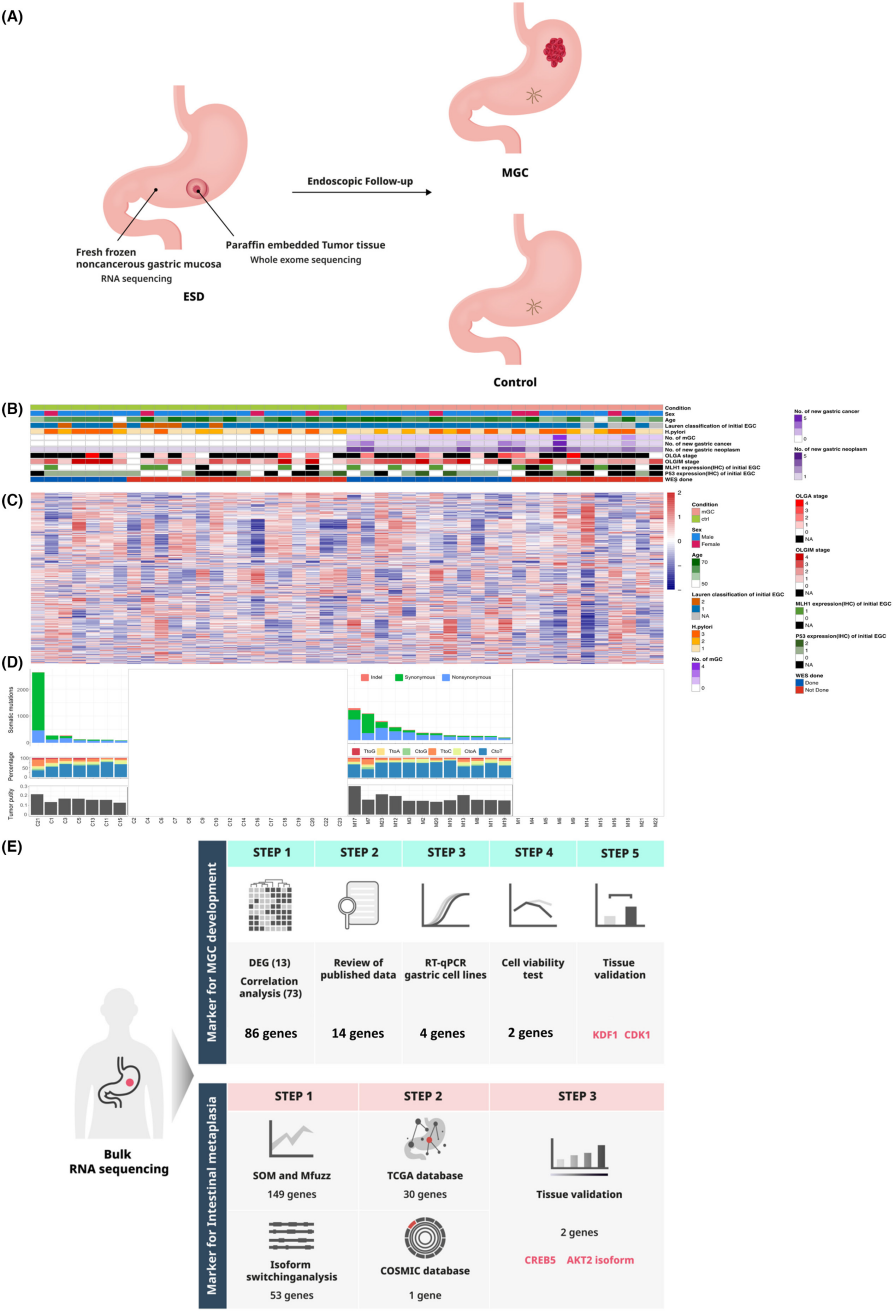
Study design and graphical summary of the clinical and sequencing data. (A) Study design. (B) Graphical summary of the clinical data (C) Heatmap of the top 1000 highly variable genes (HVGS) sorted by the standard deviation of RNA sequencing data. The expression profiles of HVGS cluster samples, and the clustering pattern implies the heterogeneous characteristics of the cohort. (D) Somatic mutation landscape. (E) The flow of target marker selection.

For biomarker discovery, patients who underwent ESD for EGC in Seoul National University Hospital between January 2013 and December 2016 were identified, of whom 23 comprised the MGC group and 23 were controls. The MGC group was defined as patients who developed new gastric cancer in an area other than the primary site at least 1 year after initial ESD. Controls were defined as patients who did not develop any additional gastric neoplasms, including carcinoma and adenoma, during a follow‐up period of at least 3 years (Figure 1A). MGC patients and controls were matched 1:1 in terms of age, sex, and *H. pylori* infection status. The inclusion criteria were patients aged ≥20 years with differentiated‐type EGC who underwent curative resection with ESD, and those from whom fresh‐frozen noncancerous gastric mucosal tissue was obtained at the time of initial EGC diagnosis. The tissue was taken from the lesser curvature of the antrum (at least two pieces), positioned at least 4 cm apart from the EGC if the EGC was located at the lesser curvature of the antrum, and all samples were stored at −80°C.^12^ Patients with a previous diagnosis of a gastric neoplasm (carcinoma and adenoma), and patients who underwent gastrectomy before ESD were excluded. For biomarker validation, patients independent from the discovery set who underwent ESD for EGC between January 2010 and December 2018 were identified with the same inclusion and exclusion criteria as in the discovery set. The validation cohort included 55 MGC patients and 125 controls.

All subjects received regular surveillance endoscopy. The number of MGC, defined as new gastric cancer detected within 1 year after ESD, was identified. The number of new gastric neoplasms (carcinoma and adenoma) were identified as well. A positive test of *H. pylori* was defined as a positive Giemsa stain or rapid urease test (CLO test) at the time of diagnosis. The patients' *H. pylori* infection status was classified as: (1) *H. pylori* test‐positive and not eradicated or eradication failed, (2) *H. pylori*‐positive and eradicated, and (3) *H. pylori*‐negative. Gastric atrophy and intestinal metaplasia, a known premalignant condition, were scored using Operative Link on Gastritis Assessment (OLGA), and Operative Link on Gastric Intestinal Metaplasia Assessment (OLGIM) stages at the time of initial diagnosis.^13^, ^14^ Stages 0–II were considered low‐risk stages, while stages III and IV were considered high‐risk stages.^15^ In addition, MutL homolog 1 (MLH1) expression from immunohistochemistry (IHC) of the ESD specimen, was identified.

The study was approved by the Institutional Review Board (IRB) of Seoul National University Hospital (H‐2003‐160‐1112).

### 2.2 Biomarker identification

#### 2.2.1 RNA sequencing quantification, differential gene expression, and correlation analysis

RNA was extracted from fresh‐frozen noncancerous gastric mucosal tissue that was obtained at the time of diagnosis using an RNA preparation kit (PureLink™ RNA Mini Kit; Thermo Fisher Scientific, Waltham, MA, USA), and cDNA was synthesized using a PrimeScript™ 1st Strand cDNA Synthesis Kit (Takara Bio Inc., Kusatsu, Japan). RNA sequencing was carried out using an Illumina HiSeq2500 sequencer by Theragen Etex Bio Institute (Suwon, Korea).

The correlation between gene expression and clinical information, including the number of new MGCs, the number of new carcinomas, the number of new neoplasms, and OLGIM stage, was investigated in the 46 samples.^13^, ^14^ A detailed protocol is available in the Appendix S1.

#### 2.2.2 Self‐organizing map analysis and Mfuzz clustering

We clustered genes using the self‐organizing map (SOM) method^16^ and the Mfuzz^17^ R package by their expression patterns according to the number of MGCs, number of new gastric carcinomas, OLGA stage, and OLGIM stage. A detailed protocol is available in the Appendix S1.

#### 2.2.3 Analysis of alternative splicing event

Gene‐level differential expression analyses might be inadequate to identify potential isoform‐level gene expression changes in the MGC group compared to controls. Therefore, a further analysis was performed to characterize specific splicing events. A detailed protocol is available in the Appendix S1.

#### 2.2.4 Module score

A detailed protocol is available in the Appendix S1.

#### 2.2.5 DNA extraction and whole‐exome sequencing analysis

The tissues used for whole‐exome sequencing (WES) consisted of 19 unmatched tumor samples from ESD specimens of the discovery cohort, of which seven were MGC patients and 12 were controls. WES could not be performed for the other samples from the discovery cohort due to low quality (most of the ESD samples had been stored for more than 5 years before this experiment) or the absence of residual tumors. A detailed protocol is available in the Appendix S1.

### 2.3 Biomarker verification

#### 2.3.1 Candidate marker expression in gastric cancer cell lines and normal gastric epithelial cell line

SNU1 (RRID:CVCL_0099), SNU5 (RRID:CVCL_0078), SNU16 (RRID:CVCL_0076), SNU216 (RRID:CVCL_3946), SNU484 (RRID:CVCL_0100), SNU601 (RRID:CVCL_0101), SNU620 (RRID:CVCL_5079), SNU638 (RRID:CVCL_0102), MKN1 (RRID:CVCL_1415), MKN45 (RRID:CVCL_0434), and AGS (RRID:CVCL_0139) human gastric cancer cells were purchased from the Korean Cell Line Bank (Seoul, Korea). HFE145 (a normal gastric epithelial cell line) was kindly provided by Dr. Hassan Ashktorab and Dr. Duane T. Smoot (Howard University, Washington, DC, USA and Meharry Medical Group, Nashville TN, USA, respectively). The expression of candidate markers in gastric cell lines was compared using real‐time quantitative polymerase chain reaction (RT‐qPCR), and markers that showed significantly higher expression in gastric cancer cell lines than in normal gastric epithelial cell lines were selected. The sequencing primers are shown in Table S1. Cells were subjected to regular mycoplasma testing, and all experiments were performed using mycoplasma‐free cells. A detailed protocol is available in the Appendix S1.

#### 2.3.2 siRNA transfection and cell viability assay

Cell viability of SNU216 was measured for 2 days using WST‐1, and biomarkers that showed reduced cell viability after small interfering RNA (siRNA) transfection (0, 25, and 50 μmol) compared to negative control siRNA were selected.^18^ The siRNA sequences used in the study are listed in Table S2. The experiment was performed three times. A detailed protocol is available in the Appendix S1.

### 2.4 Validation of target biomarkers

#### 2.4.1 Gene expression validation using RT‐qPCR and Western blot

The expression levels of target biomarkers were compared in the validation cohort between MGC patients and controls using RT‐qPCR from fresh‐frozen noncancerous gastric mucosa taken at the time of diagnosis. To optimize the predictive performance, combinations of target biomarker expression and clinical variables including age, sex, *H. pylori* infection status, and the OLGIM stage were evaluated. In addition, the expression of each biomarker was stratified and compared according to the OLGIM stage. All measurements were performed in duplicate. The sequencing primers are shown in Table S1.

Protein expression was detected using primary antibodies for target markers (CDK1 (33‐1800; Thermo Fisher Scientific, Cleveland, OH, USA), KDF1 (HPA028639; ATLAS ANTIBODIES, Stockholm, Sweden) and GAPDH (sc‐25778; Santa Cruz Biotechnology, Dallas, TX, USA)) in representative samples from the validation cohort.^18^, ^19^, ^20^ The experiment was performed three times. A detailed protocol is available in the Appendix S1.

#### 2.4.2 Immunohistochemistry

IHC staining of target markers was performed on noncancerous components of ESD specimens in the validation cohort.^20^ The primary antibodies were rabbit anti‐CDK1 (ab265590; Abcam, Cambridge, UK), and rabbit anti‐KDF1 (PA5‐55926; Invitrogen, Waltham, MA, USA). A detailed protocol is available in the Appendix S1.

### 2.5 Statistical analysis

Significantly regulated genes were identified using the Student *t*‐tests with false discovery rate correction.^21^ Receiver operating characteristic (ROC) curves were used to present the performance of individual biomarker panels. As a performance indicator, the area under the curve (AUC) values of each target marker, combinations of target markers, and combinations of target markers and clinical variables were analyzed and compared. Multivariable logistic regression analysis of the target biomarkers and clinical variables was performed to optimize the predictive model. All statistical analyses were performed using SPSS version 26 (IBM Corp., Armonk, NY, USA), GraphPad Prism version 9.4.1 (GraphPad Software, San Diego, CA, USA), and R (4.2.1., R Development Core Team, https://cran.r‐project.org/). *p*‐values <0.05 were considered statistically significant. The study was reported according to the Standards for Reporting of Diagnostic Accuracy Studies 2015 guidelines.^22^

## 3 RESULTS

### 3.1 Baseline characteristics

The baseline characteristics of patients in the discovery and validation cohorts are shown in Table 1. In the discovery cohort, an average of 1.17 MGCs (range 1–4) were newly detected in MGC patients during a median follow‐up period of 61 months (range, 28–98 months). The average numbers of new gastric neoplasms, new gastric carcinomas, and new gastric adenomas were 1.78 (range, 1–5), 1.52 (range, 1–5), and 0.26 (range, 1–4), respectively. No new gastric neoplasms were detected over a median follow‐up period of 69 months (range, 42–105 months) in the control group. In the validation cohort, an average of 1.09 MGCs (range 1–3) were newly detected in the MGC group during a median follow‐up period of 89 months (range, 24–144 months). In the control group, no new gastric neoplasms were detected over a median follow‐up period of 98 months (range, 51–129 months). In both cohorts, age, sex, and *H. pylori* infection status were well‐matched between the two groups. The follow‐up period, OLGA stage, and MLH1 expression did not exhibit significant differences between MGC and control groups. In the validation cohort, the OLGIM stage was significantly higher in MGC patients compared to the control group.

**TABLE 1:** Baseline characteristics.

Clinical characteristics	Discovery set			Validation set		
	Control group (*n* = 23)	MGC group (*n* = 23)	*p* Value	Control group (*n* = 125)	MGC group (*n* = 55)	*p* Value
Age^a^ (years), median ± SD	66 ± 5.81	66 ± 5.73	0.87	62 ± 8.38	62 ± 6.41	0.55
Sex (male), *n* (%)	19 (82.61)	19 (82.61)	1.00	89 (71.20)	43 (78.18)	0.33
Gastric neoplasm (*n*), mean (range)						
New gastric neoplasm^b^	0	1.78 (1–5)		0	1.51 (1–3)	
New gastric carcinoma	0	1.52 (1–5)		0	1.16 (0–2)	
New gastric adenoma	0	0.26 (0–1)		0	0.36 (1–3)	
MGC	0	1.17 (1–4)		0	1.09 (1–3)	
Follow‐up period, median ± SD	69 ± 9.38	61 ± 15.91	0.11	89 ± 9.15	98 ± 36.18	0.10
Helicobacter pylori status, *n* (%)						
Absent	10 (43.48)	10 (43.48)	1.00	45 (36.00)	19 (34.55)	0.34
Present, eradicated	5 (21.74)	5 (21.74)		29 (23.20)	8 (14.54)	
Present, eradication not done or failed	8 (34.78)	8 (34.78)		52 (47.20)	28 (50.91)	
OLGA stage						
Low (0–II)	8 (80.00)	7 (77.78)	0.91	45 (84.91)	14 (70.00)	0.15
High (III–IV)	2 (20.00)	2 (22.22)		8 (15.09)	6 (30.00)	
OLGIM stage						
Low (0–II)	14 (60.87)	11 (50.00)	0.46	81 (64.29)	25 (45.45)	0.02
High (III–IV)	9 (39.13)	11 (50.00)		45 (35.71)	30 (54.55)	
MLH1						
Retained expression, *n* (%)	6 (60.00)	12 (41.38)	0.31	83 (87.37)	36 (81.82)	0.39
Loss of expression, *n* (%)	4 (40.00)	17 (58.62)		12 (12.63)	8 (18.18)	

Abbreviations: MGC, metachronous gastric cancer; MLH1, MutL homolog 1; OLGA, operative link on gastritis assessment; OLGIM, operative link on gastric intestinal metaplasia assessment; SD, standard deviation.

^a^ Age at initial endoscopic submucosal dissection.

^b^ Number of new gastric neoplasm refers to the sum of new gastric adenoma and carcinoma.

### 3.2 Candidate markers for the prediction of MGC development

**FIGURE 2:**
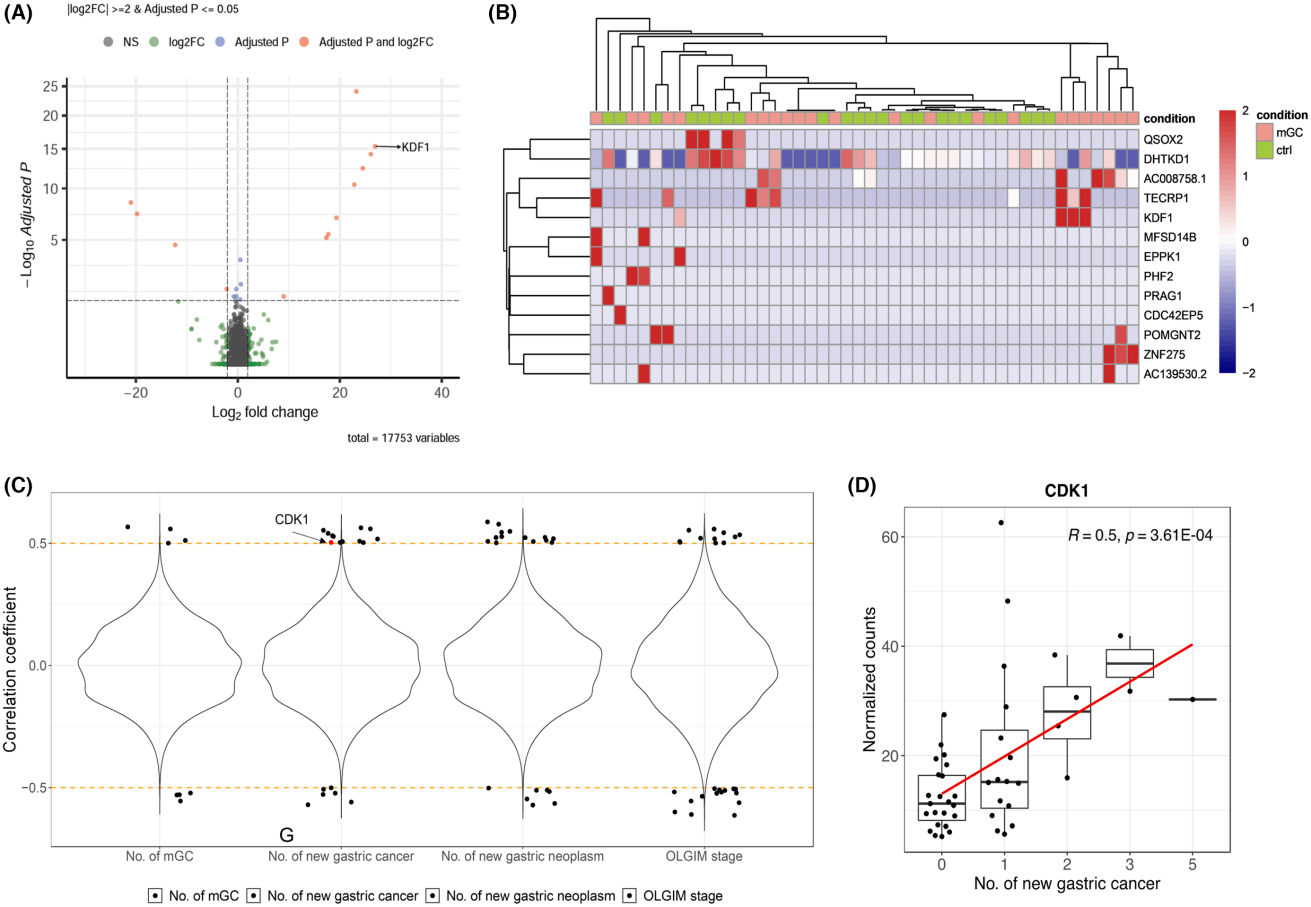
Candidate gene selection with bulk RNA sequencing data. (A) Significantly differentially expressed genes are marked as red dots in the volcano plot. (Our gene of interest gene, *KDF1*, is labeled). (B) Unsupervised hierarchical clustering of 13 significantly dysregulated genes in the control (ctrl) and MGC groups. (C) Spearman correlation analysis of gene expression by the number of MGCs, number of new gastric cancers, number of new gastric neoplasms, and OLGIM stage. Each dot represents a significant correlation, with a correlation coefficient ≥0.5 and *p*‐value <0.05. The red dot represents *CDK1*, which is our gene of interest. (D) Expression of *CDK1* by the number of new gastric cancers, with Spearman correlation coefficient and its *p*‐value. The red line indicates the regression line.

For biomarker discovery, samples in the discovery set were subjected to bulk RNA sequencing and subsequently processed through an in‐house data analysis pipeline, revealing transcriptomic heterogeneity (Figure 1B–D and Figure S1A–D). The flow of target marker selection is shown in Figure 1E. Statistically significant differentially expressed genes (DEGs) (|log_2_FC| ≥ 2 and adjusted *p*‐value ≤5 × 10^−2^) were analyzed using DESeq2, which identified 13 DEGs (nine upregulated and four downregulated genes in the MGC group) (Figure 2A,B, and Figure S2A). Correlation analysis between gene expression and clinical information (the number of new MGCs, new carcinomas, new neoplasms, and OLGIM stage) revealed 73 significantly correlated genes (Figure 2C).

From the above transcriptome analysis, 86 candidate markers were initially identified (13 from DEGs and 73 from correlation analysis). To prioritize genes, a literature review of the candidate markers was conducted to identify genes associated with cell proliferation, tumor growth, invasion, and prognosis, and 14 markers were selected for additional verification (*KDF1*, *EPPK1*, *QSOX2*, *DHTKD1*, *MAP1B*, *CENPJ*, *E2F3*, *TMED2*, *TM9SF4*, *COL3A1*, *CCDC116*, *TAL1*, *CDK1*, and *VPS13B*). Among these markers, keratinocyte differentiation factor 1 (*KDF1*) and cyclin‐dependent kinase 1 (*CDK1*), which were selected for detailed investigations in the laboratory experiments described later, are illustrated in Figure 2A,C,D.

### 3.3 Identification of candidate markers highly expressed in gastric cancer cell lines and associated with cell viability

**FIGURE 3:**
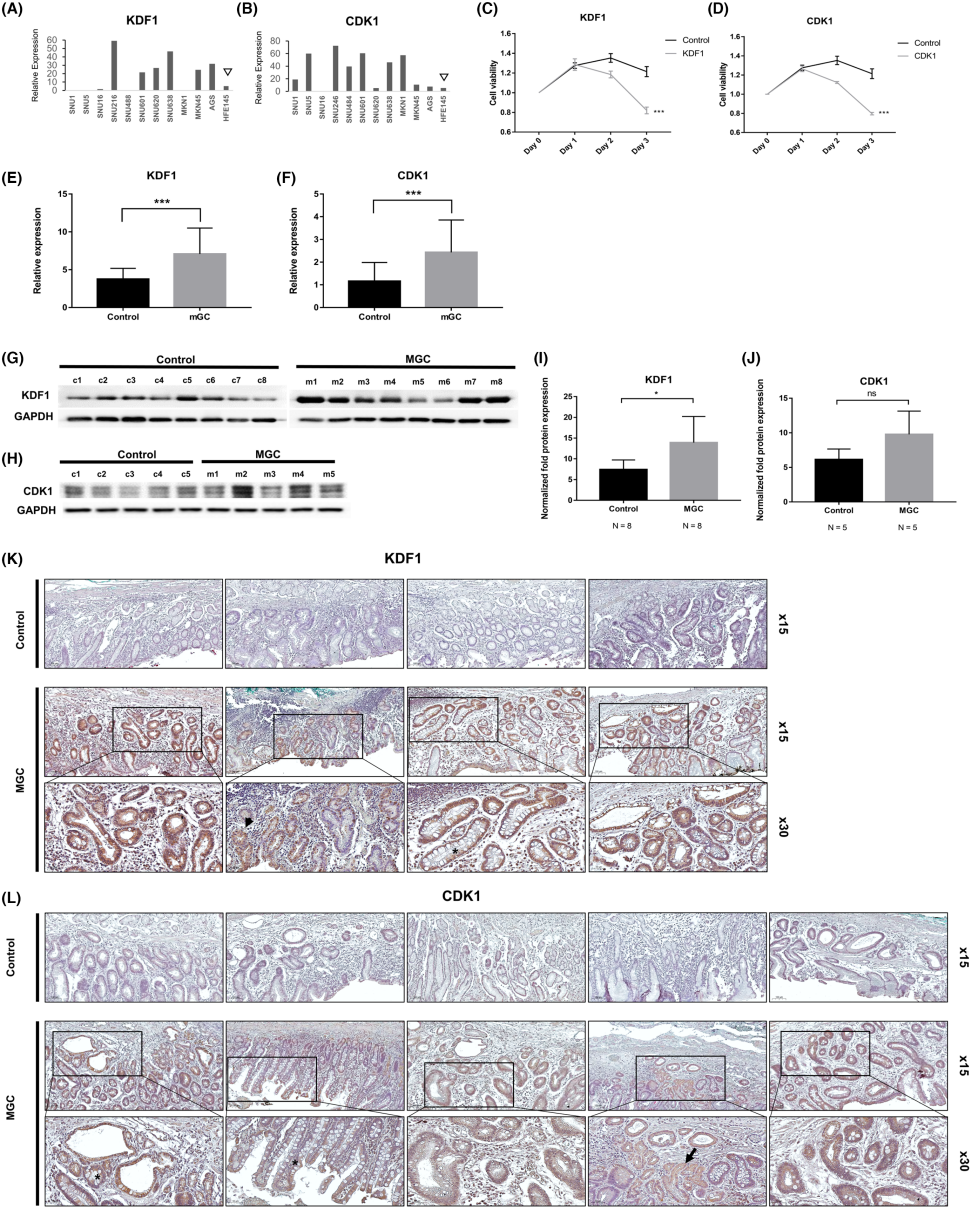
Selection and validation of the target markers, *KDF1* and *CDK1*. (A, B) Relative expression of *KDF1* and *CDK1* in various gastric cancer cell lines and a normal gastric epithelial cell line (HFE145) (arrowhead). (A) *KDF1* and (B) *CDK1* showed increased expression in various gastric cancer cell lines compared to HFE145 (intestinal type: SNU216, MKN1, AGS; diffuse type: SNU1, SNU5, SNU16, SNU488, SNU601, SNU620, SNU638, MKN45; normal gastric epithelial cell line: HFE145). (C, D) Cell viability test using siRNA. Knockdown of (C) *KDF1* and (D) *CDK1* using specific siRNAs resulting in reduced cell viability in a gastric cancer cell line (SNU216). (E–L) Validation of *KDF1* and *CDK1*. (E, F) RT‐qPCR in fresh‐frozen normal gastric epithelial tissue of MGC patients and controls in the validation set measured expression levels of KDF1 and CDK1. (E) *KDF* and (G) *CDK1* showing significantly increased expression in MGC patients compared to controls. (G, H) Western blot of KDF1 and CDK1 in MGC patients and controls. (G) Representative western blot image of KDF1 in MGC and control samples from three independent experiments. (H) Representative western blot image of CDK1 in MGC and control samples from three independent experiments. (I, J) Intensity analysis of the Western blots of (I) KDF1 and (J) CDK1 in MGC patients and controls, normalized and displayed in bar diagrams. (K, L) Immunohistochemical staining of (K) KDF1 and (L) CDK1 in noncancerous gastric epithelial tissues. Representative samples were obtained from MGC patients and controls from the validation cohort. (K) Immunohistochemical staining revealed weak KDF1 expression in the normal gastric epithelial tissue of controls and high KDF1 expression in normal tissues of MGC patients. In the MGC patients, the normal gastric epithelium (arrow) and areas with intestinal metaplasia (*) both showed increased expression of KDF1. Upper and middle panels: Magnification ×15; lower panels: Magnification ×30. Data are representative of three independent experiments. (L) Weak CDK1 expression in normal gastric epithelial tissue of controls and high CDK11 expression in normal tissues of MGC patients. In the MGC patients, the normal gastric epithelium (arrow) and areas with intestinal metaplasia (*) both showed increased expression of CDK1. Upper and middle panels: Magnification ×15; lower panels: Magnification ×30. Data are representative of three independent experiments. *p < 0.01 ***p < 0.0001.

Among the 14 candidate markers identified from the discovery set for MGC prediction, four genes that showed higher expression in gastric cancer cell lines than in the normal gastric epithelial cell line (HFE145) were selected (*KDF1*, *E2F3*, *CDK1*, and *DHTKD1*) (Figure 3A,B, and Figure S3).

For the cell viability assay, SNU216 was chosen, which commonly showed increased expression of the candidate markers in the RT‐qPCR assay. After the knockdown of the four candidate genes with siRNA transfection, *KDF1* and *CDK1* showed reduced expression and the knockdown of the two genes efficiently reduced cell viability (*KDF1*, 67.56 ± 2.77%; *CDK1*, 65.72 ± 3.81%, all *p <* 0.05) (Figure 3C,D, and Figure S4).

### 3.4 Overexpression of *KDF1* and *CDK1* in MGC patients in the validation cohort

To validate the two putative markers (*KDF1* and *CDK1*), RT‐qPCR was performed in a validation cohort of 180 patients. The expression of *KDF1* and *CDK1* were significantly higher in MGC patients than in controls (*p <* 0.001 in both) (Figure 3E,F). Western blot showed higher expression of CDK1 and KDF1 in MGC patients than in controls (KDF1, *p* = 0.021; CDK1, *p* = 0.056) (Figure 3G–J). IHC staining revealed stronger expression of both markers in the normal tissues of MGC patients than in controls. (Figure 3K,L). The relative expression of KDF1 and CDK1 according to the OLGIM stage showed no significant difference (Figure S5).

### 3.5 Markers representing gastric atrophy and intestinal metaplasia

**FIGURE 4:**
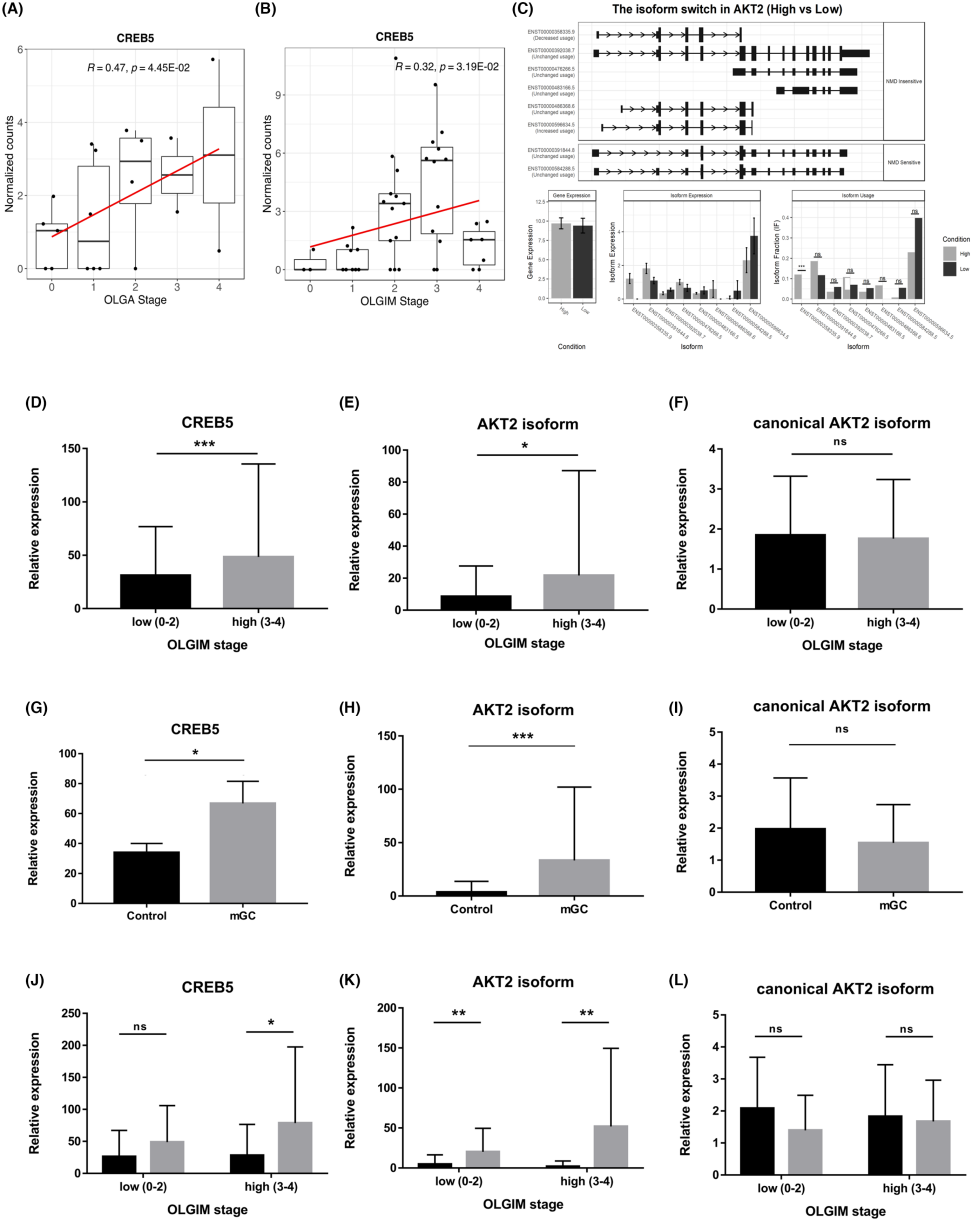
Markers associated to gastric atrophy and intestinal metaplasia. (A, B) Expression of *CREB5* by the (A) OLGA stage and (B) OLGIM stage in the bulk RNA sequencing data from the discovery cohort with Spearman correlation coefficient and its *p* value. The red line indicates the regression line. (C) Isoform switching analysis of *AKT2*. The isoform structures of *AKT2* along with the concatenated annotations (including transcript classification, ORF, coding potential, and NMD sensitivity) are shown in the upper panel. Differential gene expression and isoform fraction (dIF) between the OLGIM high (*n* = 20) and OLIGM low (*n* = 3) group were shown in the lower panel (** dIF >0.1 and *p* value <0.001.) (D–L) Expression levels of *CREB5*, *AKT2* isoform (ENST00000358335.9), and canonical isoform AKT2 in the validation cohort according to OLGIM stage and MGC development. (D–F) Expression levels of (D) *CREB5*, (E) *AKT2* isoform (ENST00000358335.9), and (F) canonical isoform *AKT2* measured by RT‐qPCR in fresh‐frozen normal gastric epithelial tissue of the validation set, and stratified by OLGIM stage low (0–II) to high (III–IV). *CREB5* and *AKT2* isoform (ENST00000358335.9) shows good correlation with OLGIM stage while canonical isoform *AKT2* does not show such significance. (G–I) Expression levels of (G) *CREB5*, (H) *AKT2* isoform (ENST00000358335.9), and (I) canonical isoform *AKT2* measured by RT‐qPCR in fresh‐frozen normal gastric epithelial tissue of MGC patients and controls in the validation cohort. (G) *CREB5* and (H) *AKT2* isoform (ENST00000358335.9) showing significantly increased expression in MGC patients compared to controls while (I) canonical isoform *AKT2* does not show such significance. (J–L) Expression levels of (J) *CREB5*, (K) *AKT2* isoform (ENST00000358335.9), and (L) canonical isoform *AKT2* measured by RT‐qPCR in fresh‐frozen normal gastric epithelial tissue of MGC patients and controls in subgroups with low OLGIM stage (0–II) patients and high OLGIM stage (III–IV) patients in the validation cohort. (J) In OLGIM high (III–IV) patients, *CREB5* showed increased expression in MGC patients compared to controls, while in OLGIM low (0–II) patients, no significant difference was noticed. *p < 0.01, **p < 0.001, ***p < 0.0001.

To model gene expression patterns correlated with the clinical information, the SOM and Mfuzz techniques were applied. Four clusters (SOM clusters 3 and 9, Mfuzz clusters 6 and 13) showed elevated gene expression as the OLGA stage increased (Figure S2B,C). Among them, the expression of CAMP‐responsive element binding protein 5 (CREB5), which is previously known to be associated to cancer invasion, metastasis, and poor survival, significantly increased with both the OLGA and OLGIM stages (Figure 4A,B).^23^, ^24^ Detailed results are available in the Appendix S1.

Isoform switching analysis was performed to characterize specific splicing events and identify potential isoform‐level gene expression changes. No significant association was noticed in isoform‐level gene expression with MGC development. Additional isoform switching analysis was performed according to the severity of gastric mucosal atrophy and intestinal metaplasia, which identified 53 genes with differential transcript usage in the high‐risk OLGIM group (stages III–IV) than in the low‐risk OLGIM group (stages 0–II).^10^ After the manual curation of known cancer genes from the COSMIC Cancer Census Tier 1, all genes except for *AKT* serine/threonine kinase 2 isoform (ENST00000358335.9, hereafter referred to as *AKT2* isoform) were eliminated (Figure 4C).^25^ No difference was noticed in the whole‐gene expression of *AKT2* (canonical isoform) between the high‐risk and low‐risk groups, meaning that the previous DEG analysis would not pick up any changes in *AKT2* (Figure 4C).

### 3.6 *CREB5* and *AKT2* isoform are overexpressed in patients with severe intestinal metaplasia in the validation cohort

*CREB5* and *AKT2* isoform were validated according to the OLGIM stage and the occurrence of MGC. Both *CREB5* and the *AKT2* isoform showed significantly higher expression in the high‐risk OLGIM group (stages III–IV) than in the low‐risk OLGIM group (stages 0–II) (*p <* 0.001 in both) (Figure 4D,E, Figure S6A,B). Also, they showed a higher expression level in MGC patients compared to controls (*CREB5*, *p* = 0.015; *AKT2* isoform, *p* < 0.001) (Figure 4G,H). In a subgroup analysis according to MGC development in low‐risk OLGIM patients and high‐risk OLGIM patients, *CREB5* showed higher expression in MGC patients than in controls among high‐risk OLGIM patients, while no significant difference was shown between MGC patients and controls among low‐risk OLGIM patients (Figure 4J,K). The expression of the canonical isoform of *AKT2* in the validation cohort showed no significant difference according to either OLGIM stage or MGC development (low‐risk OLGIM vs. high‐risk OLGIM, *p* = 0.75; MGC vs. control, *p* = 0.12) (Figure 4F,I, L and Figure S6C).

### 3.7 Performance of target biomarkers

**FIGURE 5:**
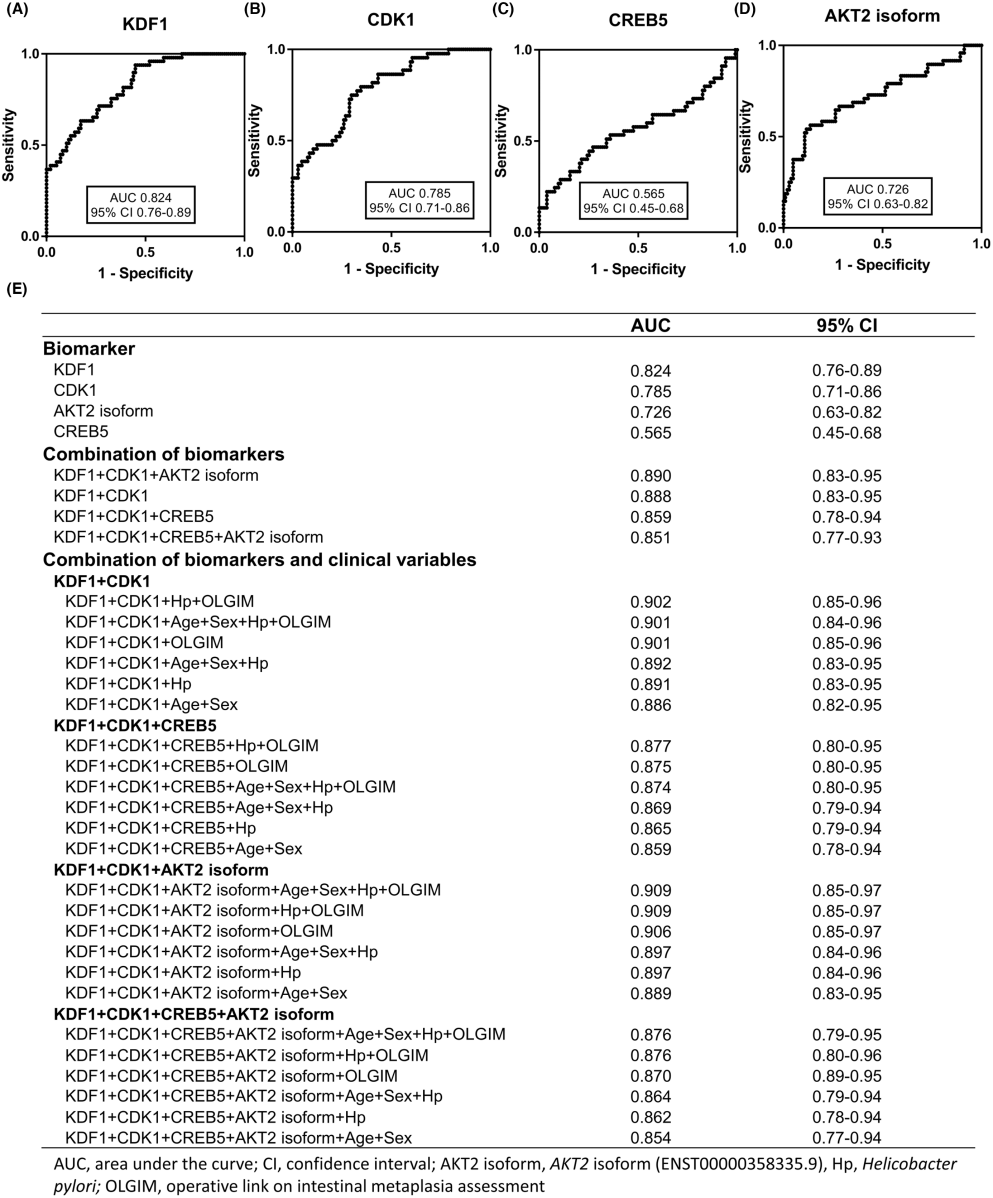
Accuracy of biomarkers in the validation cohort. (A–D) Receiver operating characteristic (ROC) curves for *KDF1* (A), *CDK1* (B), *CREB5* (C), and *AKT2* isoform (ENST00000358335.9) (D). The area under the curve (AUC) was used to determine the accuracy of MGC prediction. (E) Combinations of biomarkers with clinical information including age, sex, *H. pylori* infection status, and OLGIM stage with the optimal AUC for predicting MGC development (listed in order of highest to lowest AUC values according to each biomarker, combination of biomarkers, and combination of biomarkers and clinical variables).

The performance of the four biomarkers (*KDF1*, *CDK1*, *CREB5*, and *AKT2* isoform) was analyzed using AUC values (Figure 5). The AUC of each biomarker for MGC prediction was 0.82 (95% CI, 0.76–0.89; *p <* 0.001) for *KDF1*, 0.79 (95% CI, 0.71–0.86; *p <* 0.001) for *CDK1*, 0.57 (95% CI, 0.45–0.68; *p =* 0.209) for *CREB5*, and 0.73 (95% CI, 0.63–0.82; *p <* 0.001) for *AKT2* isoform (Figure 5A–D).

Figure 5E shows the AUC values of each biomarker, the combinations of biomarkers, and the combination of biomarkers and clinical variables for MGC prediction. The accuracy of MGC prediction was improved by combining biomarkers and including patients' clinical information such as age, sex, *H. pylori* infection state, and OLGIM stage. The highest value of 0.909 was observed for the combination of three biomarkers (*KDF1*, *CDK1*, and *AKT2* isoform) with clinical variables (Figure 5E). The combination of *KDF1* and *CDK1* with clinical variables also showed a high AUC value (0.901).

After identifying target markers, the module score combining the target markers from the transcriptome data of the discovery set was calculated for additional analysis. The MGC group displayed significantly higher gene module scores for *KDF1*, *CDK1*, and *CREB5* than the control group, demonstrating that these three genes could serve as effective biomarkers for the prediction of MGC (Figure S7). *AKT2* isoform could not be included since DEG analysis would not detect any changes to *AKT2*, as discussed above.

### 3.8 Mutational burden is increased in tumor tissue of MGC patients

There were significant differences in the number of somatic variants between control and MGC groups without a hypermutator (Wilcoxon *p*‐value = 3.20 × 10–2, *p*‐value with hypermutator = 1.67 × 10^−2^) (Figure 1D, Figure S8). In two MGC samples, two independent recurrent somatic mutations were observed in vascular endothelial zinc finger 1 (*VEZF1*), which is a cancer‐related gene^26^ (Figure S8C and Table S3). Details are presented in the Appendix S1.

## 4 DISCUSSION

A molecular analysis of gastric cancer revealed its complex landscape and heterogeneity in genetic and epigenetic alterations.^27^ The molecular features and carcinogenesis of EGC still remain largely unknown.^28^ For patients treated with ESD who have a potential risk of recurrence in the remnant stomach, the landscape of the remnant gastric mucosa, as well as the initial tumor itself, should be considered since new cancers commonly arise in areas other than the primary site. In light of this concern, both the noncancerous mucosa and the tumor tissue obtained at the time of initial diagnosis of EGC patients were evaluated in this study. To the best of our knowledge, this is the first study to reveal biomarkers for MGC development using transcriptome and exome sequencing data from noncancerous gastric epithelial tissue and tumor tissue at the time of initial diagnosis. Previously, Asada et al. demonstrated the usefulness of an epigenetic marker for predicting MGC.^29^ Sakuta et al. analyzed EGC and adjacent normal mucosa for somatic variants and reported that a higher mutational burden was associated with MGC development.^30^ However, functional analysis and marker validation were limited in the previous studies.

The two most important biomarkers identified in this study are *KDF1* and *CDK1*. *KDF1* is a key regulator of epidermal proliferation and differentiation.^31^ Previous studies regarding the role of *KDF1* in tumorigenesis showed inconsistent results and no studies have reported the role of KDF1 in gastric cancer. In ovarian cancer, KDF1 was reported to play an oncogenic role, while it served as a tumor suppressor in renal cell carcinoma.^32^, ^33^ CDK1, a key regulatory enzyme that controls cell cycle transition, is known to be associated with cancer proliferation and rapid tumor growth.^34^, ^35^ It is known to be linked to the progression and poor prognoses in multiple types of cancer, including colon cancer, pancreatic cancer, lung cancer, and breast cancer.^36^, ^37^, ^38^, ^39^ Regarding gastric cancer, Zhu et al reported the role of CDK1 in gastric cancer development by demonstrating that CDK1 bridges NF‐κB and β‐catenin signaling in response to *H. pylori* infection.^40^ In our study, higher levels of CDK1 and KDF1 expression in noncancerous gastric mucosa were associated with an increased risk of future cancer development, suggesting that elevated expression of these markers may indicate a precancerous condition and play a role in gastric tumorigenesis. Cell viability was reduced with the knockout of KDF1 and CDK1, supporting their role as oncogenes and potential involvement in promoting gastric cancer progression.

The uncharacterized isoform switch in *AKT2* merits attention, as it has been shown that the *AKT2* isoforms have been suggested to play a specific role in cancer progression.^41^, ^42^, ^43^ A recent study reported that alternative *AKT2* splicing could produce a protein that lacks the hydrophobic motif regulatory region, which leads to the expression of deregulated forms of AKT2.^44^ The upregulated usage of *AKT2* isoform in the high‐risk OLGIM group lacked the kinase domain present in the canonical *AKT2* isoform, which could indicate that increased levels of the isoform dysregulated signaling pathways, facilitating tumor progression. This result was validated with isoform‐specific primer pairs in RT‐qPCR.

CREB5 is known to be upregulated in various cancers, and promote invasiveness and metastasis.^24^ Interestingly, *CREB* is known to be a key negative regulator of carbonic anhydrase IX (*CA9*), which is downregulated in gastric cancer.^45^ In our study, *CREB5* had a relatively low AUC value for MGC prediction compared to other markers. It seems to play a more dominant role in reflecting the severity of intestinal metaplasia rather than MGC prediction.

According to the multistep model of gastric carcinogenesis, known as the “Correa cascade,” gastric cancer is generally preceded by a sequence of precancerous lesions, such as atrophic gastritis, intestinal metaplasia, and dysplasia.^46^ In the present study, *KDF1* and *CDK1*, markers for MGC development, did not show significant associations with the OLGIM stage in the validation cohort. This suggests that *KDF1* and *CDK1* may be markers independent of intestinal metaplasia severity, and could play a distinct role in MGC progression apart from the gastric precancerous cascade, potentially being more linked to cancer development rather than premalignant changes. On the contrary, the expression of *CREB5* and *AKT2* isoform significantly increased as the OLGIM stage progressed. *CREB5* and *AKT2* isoform were markers that showed higher expression in the MGC group than the controls in the validation cohort, but were not initially identified as meaningful markers associated with MGC development in the discovery cohort. This suggests that *CREB5* and *AKT2* isoform could be indirectly associated with MGC development through mechanisms that promote intestinal metaplasia. In a subgroup analysis, the high‐risk OLGIM group showed significantly higher *CREB5* expression in MGC patients compared to controls, whereas the low‐risk OLGIM group showed no significant difference in *CREB5* expression between MGC patients and controls. This suggests that *CREB5* may be more closely associated with the development of intestinal metaplasia, rather than being directly linked to MGC development.

Meanwhile, CREB5 and AKT2 isoform may have clinical relevance for patients with gastric premalignant lesions. Considering that assessing the severity of intestinal metaplasia (OLGIM stage) typically requires multiple biopsies from at least five different sites in the stomach,^13^, ^14^ the finding that biomarkers from a single site (antrum lesser curvature) correlates well with the OLGIM stage indicates that these markers can be applied in patients with intestinal metaplasia both to assess the severity of metaplasia and predicting the risk of MGC.

WES analysis showed significant differences in the number of somatic variants between the control and MGC groups, which is consistent with the previous study.^30^

This study has several limitations. First, since this study was only conducted in patients who matched the strict inclusion and exclusion criteria, and whose noncancerous mucosal biopsy samples were well‐preserved in a deep freezer for years, obtaining a large sample size was difficult. Second, selection bias may have existed due to the retrospective nature of the study, although well‐known risk factors such as age, sex, and *H. pylori* were matched between the two groups. The variability in the expression levels of biomarkers could be influenced by genetic diversity among the patient population, environmental factors, and lifestyle differences that were not accounted for in this study. The heterogeneity of the gastric mucosal environment and the influence of other underlying gastric conditions could also impact the expression of these biomarkers. Third, during WES, more than half of the ESD samples in the discovery set could not be further analyzed due to poor conditions (most of the ESD samples used in this study were stored for years because long‐term follow‐up was essential for categorizing the metachronous cancer groups) or the absence of residual tumors. Fourth, the issue of patchiness should be considered. Gastric mucosal biopsies were taken from the antrum, which may not be sufficient to represent the landscape of the entire remnant gastric mucosa. However, considering that obtaining biopsies from multiple sites can be challenging in real‐world practice, a single representative site may be more practical for the clinical implementation of our findings. This is particularly relevant given that the expression of *CREB5* and *AKT2* isoform from the lesser curvature of gastric antrum (single‐site) correlated well with the OLGIM stage, a well‐established staging system that combines intestinal metaplasia severity of the gastric antrum, corpus, and incisura angularis. Lastly, this study was developed and validated entirely in an Asian population where the incidence of gastric cancer is high. Further studies in other populations are warranted.

This is the first study to develop and validate biomarkers for MGC development that showed high accuracy in predicting MGC. *KDF1* and *CDK1* were identified as predictive markers for MGC development in EGC patients who undergo ESD. *CREB5* and *AKT2* isoform indicate the severity of intestinal metaplasia, and also serve as markers for MGC development. These biomarkers could be incorporated into clinical practice to stratify EGC patients based on their risk of developing MGC, enabling tailored surveillance strategies, and potentially improving patient outcomes through early detection. Future research should focus on elucidating the molecular mechanisms underlying these biomarkers, validating their predictive value in broader populations, and integrating them with other biomarkers to enhance risk prediction models.

The assessment of biomarkers on noncancerous gastric mucosa at the time of initial diagnosis is a useful method of predicting MGC development in EGC patients who undergo endoscopic resection. These findings provide new insights into the mechanisms of tumorigenesis and MGC development and suggest a potential role of these biomarkers in gastric cancer screening and treatment.

## AUTHOR CONTRIBUTIONS

**Bokyung Kim:** Conceptualization (equal); data curation (equal); formal analysis (equal); investigation (equal); methodology (equal); resources (equal); validation (equal); visualization (equal); writing – original draft (lead); writing – review and editing (equal). **Harim Chun:** Conceptualization (equal); data curation (equal); formal analysis (equal); investigation (equal); methodology (equal); visualization (equal); writing – original draft (lead); writing – review and editing (equal). **Jongwon Lee:** Conceptualization (equal); formal analysis (equal); investigation (equal); visualization (equal); writing – original draft (equal); writing – review and editing (equal). **Miree Park:** Conceptualization (equal); data curation (equal); formal analysis (equal); investigation (equal); methodology (equal); resources (equal); validation (equal); visualization (equal); writing – original draft (equal); writing – review and editing (equal). **Yoonjin Kwak:** Conceptualization (equal); investigation (equal); validation (equal); visualization (equal); writing – review and editing (equal). **Jung Mogg Kim:** Conceptualization (equal); funding acquisition (equal); writing – review and editing (equal). **Sang Gyun Kim:** Conceptualization (equal); data curation (equal); funding acquisition (equal); methodology (equal); resources (equal); writing – review and editing (equal). **Ji Kon Ryu:** Conceptualization (equal); funding acquisition (equal); methodology (equal); writing – review and editing (equal). **Jungmin Choi:** Conceptualization (equal); data curation (equal); formal analysis (equal); funding acquisition (equal); investigation (equal); methodology (equal); project administration (equal); supervision (equal); visualization (equal); writing – original draft (equal); writing – review and editing (equal). **Soo‐Jeong Cho:** Conceptualization (equal); data curation (equal); formal analysis (equal); funding acquisition (equal); investigation (equal); methodology (equal); project administration (equal); resources (equal); supervision (equal); validation (equal); visualization (equal); writing – original draft (equal); writing – review and editing (equal).

## FUNDING INFORMATION

The National Research Foundation of Korea (#NRF‐2022R1A2B5B01001430), The Korean College of Helicobacter and Upper Gastrointestinal Research Foundation (KCHUGR – 202002001), and the SNUH research fund (#03‐2022‐0140, #03‐2020‐0370) supported Soo‐Jeong Cho. The Korean College of Helicobacter and Upper Gastrointestinal Research Foundation (KCHUGR‐202102001) supported Sang Gyun Kim. The Seoul National University College of Medicine Research foundation (800‐20220338) supported Ji Kon Ryu. The National Research Foundation of Korea (#NRF‐2022R1A4A2000827) supported Jungmin Choi. The Basic Science Research Program through the National Research Foundation of Korea (NRF) funded by the Ministry of Education, Science and Technology (MEST) (NRF‐2021R1F1A1045550) supported Jung Mogg Kim.

## CONFLICT OF INTEREST STATEMENT

The authors declare that they have no conflict of interest.

## ETHICS STATEMENT

The study was approved by the Institutional Review Board (IRB) of Seoul National University Hospital (H‐2003‐160‐1112). All procedures followed were in accordance with the ethical standards of the responsible committee on human experimentation (institutional and national) and with the Helsinki Declaration of 1964 and later versions. Informed consent to be included in the study, or the equivalent, was obtained from all patients.

## Supporting information


Appendix S1.

## Data Availability

Data generated or analyzed for this study are included in this published article and its Appendix S1. Data are also available from the corresponding author upon reasonable request.
