# Supplementary material for: Predictive biomarkers for metachronous gastric cancer development after endoscopic resection of early gastric cancer
Source: Cancer Med. 2024 Aug 22;13(16):e70104. doi: 10.1002/cam4.70104 (PMC11339598; doi:10.1002/cam4.70104)
Supplement: Supplementary file 1 — Appendix S1. [file CAM4-13-e70104-s001.docx]

**Supplementary Data**

1. **Supplementary Methods**
   1. RNA sequencing quantification, differential gene expression and correlation analysis
   2. SOM analysis and Mfuzz clustering
   3. Analysis of alternative splicing event
   4. Module Score
   5. DNA extraction and Whole Exome Sequencing Analysis
   6. Candidate marker expression in gastric cancer cell lines and normal gastric epithelial cell line
   7. siRNA (small interfering RNA) transfection and cell viability assay
   8. Gene expression validation using RT-qPCR and Western blot
   9. Immunohistochemistry

1. **Supplementary Results**
   1. Markers representing gastric atrophy and intestinal metaplasia
   2. Mutational burden is increased in tumor issue of MGC patients
2. **Supplementary Figures**
   1. Supplementary Figure 1. Results of bulk RNA sequencing.
   2. Supplementary Figure 2. Gene expression of 13 DEGs and results of SOM/Mfuzz analysis
   3. Supplementary Figure 3. Relative expression of candidate genes in various gastric cancer cell lines and normal gastric epithelial cell lines.
   4. Supplementary Figure 4. Knockdown of candidate genes with siRNA transfection (0, 25, and 50 μmol)
   5. Supplementary Figure 5. Expression of *KDF1* and *CDK1* according to OLGIM stage.
   6. Supplementary Figure 6. Expression of *CREB5*, *AKT2* isoform (ENST00000358335.9) and canonical isoform AKT2 according to OLGIM stage.
   7. Supplementary Figure 7. Module scores consisted of KDF1, CDK1, and CREB5 for all samples and scores are significantly different between control and mGC groups by the Wilcoxon test.
   8. Supplementary Figure 8. Somatic variants, mutational signatures and frequently detected cancer genes.
3. **Supplementary Tables**
   1. Supplementary Table 1. Detail of primer sequence for Q-PCR
   2. Supplementary Table 2. Detail of sequence for siRNA
   3. Supplementary Table 3. Recurrent somatic mutations from MGC (red) and control (blue) patients.
   4. Supplementary Table 4. Survival analysis based on the Kaplan-Meier method using The Cancer Genome Atlas (TCGA) of SOM and Mfuzz results.
   5. Supplementary Table 5. Variants stats.
4. **References of supplementary data**
5. **Supplementary methods**
   1. **RNA sequencing quantification, differential gene expression and correlation analysis**

Paired-end RNA sequencing reads from 46 patients were quantified onto human reference genome GRCh38^1^ using Salmon.^2^ 46 quantified transcript abundance files were imported by the tximport^3^ for the differentially expressed gene analysis. All imported data were used to generate a DESeqDataSet by using the DESeqDataSetFromTximport function from the DESeq2 R package.^4^ Before the differential gene expression analysis, ComBAT-seq^5^ was used for adjusting the batch effect by the *H. pylori* infection status and an adjusted count data was generated accordingly. Also, surrogate variables were estimated with the surrogate variable analysis (SVA) R package^6^ to adjust unwanted variation. Differential expression analysis was conducted by using a single function, DESeq from DESEq2 and the p-values were corrected for the multiple testing using the Benjamini-Hochberg^7^. |Log_2_ Fold Change| $\geq$ 2 and adjusted *p*-values < 0.05 were used as criteria for defining statistically significant differentially expressed genes (DEGs). Principal Component Analysis (PCA) plot was generated by using the plotPCA (returnData=TRUE to customize the plot) of DESeq2. A volcano plot to visualize the negative binomial test result was generated with the EnhancedVolcano R package^8^ Count data was variance stabilizing transformed by the vsd function of DESeq2. 46 samples were unsupervised hierarchical clustered by the ward.D2 linkage method^9^ and visualized by using the pheatmap R package.^10^ Highly variable genes were selected by the standard deviation of the gene expression.

For correlation analysis, the Spearman correlation test was conducted between the normalized count of each gene and the above clinical information using the cor.test(method = ’spearman’) function in R. The significance threshold was set to |correlation coefficient| ≥ 0.5 and p-values < 0.05.

- 1. **SOM analysis and Mfuzz clustering**

Normalized counts data of 33,335 genes were clustered into 4X4 hexagonal SOM clusters by its expression patterns using the Kohonen R package.^11^ SOM clusters were trained 100,000 times and the results were visualized by the codes plot rendered by the line. Unsupervised clustering was conducted using the Mfuzz R package which implements a fuzzy c-means algorithm.^12^ Normalized count data of 33,335 genes were standardized using standardise function, the optimal fuzzifier *m* was estimated using mestimate function, and clusters were estimated by using a single function mfuzz of Mfuzz. With every 16 clusters from SOM and Mfuzz, clusters showing the increasing and decreasing patterns were selected. Overlapping genes were discovered with selected clusters showing increasing patterns from SOM and Mfuzz and those genes were tested to whether they are positively correlated with the variables. The significance cutoff was set to correlation coefficient $\geq$ 0.4 and *p*-values < 0.05. Survival analysis was conducted with remained significant genes using Kaplan Meier Plotter and genes with log-rank *p* < 0.05 were regarded as significant.^13,14^ Overlapping genes from clusters showing decreasing patterns from SOM and Mufzz also were undergone a similar procedure above except that the significant cutoff of correlation analysis was set to correlation coefficient $\leq$ -0.4 and *p*-values < 0.05.

- 1. **Analysis of alternative splicing event**

Differential transcript usage (DTU) analysis was performed using the R package IsoformSwitchAnalyzeR.^15^ Following approaches, Salmon quantification tables were imported using tximeta function to create a swtichAnalyzeRlist object. Analysis of alternative splicing event was evaluated using isoformSwitchAnalysisCombined function with default parameter except following options; alpha = 0.05, dIFcutoff = 0.1, switchTestMethod = DEXSeq. Significantly changed isoforms were selected and visualized with the switchPlot function.

- 1. **Module Score**

Module score was calculated with R script implementing the algorithm AddModuleScore function used from the Seurat R package.^16^ It 1) calculates the average expression of all genes from all samples, 2) divides all genes into the predefined number (default: 25) of bins, 3) randomly selects the predefined number (default: 100) of control genes from the same bin as the genes in the module (or signature) belong to, 4) calculates the average expression of the gene-set of the module and the randomly selected control genes, and 5) subtracts the average expression of signature genes and control genes.

- 1. **DNA extraction and Whole Exome Sequencing Analysis**

Carcinoma components from ESD specimen were separately microdissected from the formalin-fixed paraffin-embedded (FFPE) ESD specimen slides. DNA was isolated using GeneRead DNA FFPE kit (Qiagen, Hilden, Germany), according to the manufacturer’s protocol. For library preparation, SureSelect XT HS Reagent Kit (Agilent, Santa Clara, CA, USA) was used following the manufacturer’s instructions. WES procedures including exome capturing and sequencing were performed at Theragen Etex Bio Institute (Suwon, Korea). WES data were processed with the GATK Best Practices Workflow.^17,18^ Paired-end reads were aligned to GRCh37 using BWA-MEM.^19^ Somatic variants were called with the Mutect2^20^ using the Panel of Normals (PON). Called somatic variants underwent manual visual inspection and were annotated using Annovar.^21^ Somatic mutation signatures were analyzed with Deconstrucsig^22^ and mutations of cancer genes were visualized with oncoPrint function of ComplexHeatmap.^23^

- 1. **RNA extraction and real-time quantitative polymerase chain reaction (RT-qPCR) from gastric cancer cell lines and normal gastric epithelial cell line**

All cells were cultured in RPMI-1640 medium supplemented with 10% heat-inactivated fetal bovine serum (FBS), penicillin, and streptomycin and were maintained in 5% CO2 at 37℃. RNA was extracted from gastric cancer cell lines and HFE145 using an RNA prep kit (PureLink™ RNA Mini Kit; Thermo Fisher Scientific, Waltham, MA, USA), and cDNA was synthesized using a PrimeScript™ 1st Strand cDNA Synthesis Kit (Takara Bio Inc., Kusatsu, Japan). For RT-qPCR, cDNA was amplified using Ampigene qPCR Green mix Hi-ROX (Enzo Life Sciences, Farmingdale, NY, USA) with StepOne Real-Time PCR System (Applied Biosystems, CA, USA).

- 1. **siRNA (small interfering RNA) transfection and cell viability assay**

SNU216 cells were plated on 60mm culture dish and cultured in serum-free medium at 37℃ until to grow to 70% confluence. Next day, the cells were transfected with target siRNA and negative control (AccuTarget™ Negative Contol siRNA) siRNA for 24h according to maufacturer’s protocol).^24^ After siRNA transfected SNU216 (5 X 103 per well) cell seeded in 96-well culture plate in RPMI-1640 with 0.1% FBS and incubated overnight, the cell viability was determined for 2 days using WST-1 ((4-[3-(4-iodophenyl)-2-(4-nitrophenyl)-2H-5-tetrazolio]-1,3-benzene disulfonate; Takara Bio Inc., Japan) according to the manufacturer’s protocol

- 1. **Western blot analysis from normal gastric epithelial tissue from MGC patients and Controls**

For Protein extraction, patients’ fresh-frozen normal gastric epithelial tissue ware homogenized using 10X RIPA buffer (#9806; Cell Signaling Technology, Danvers, MA, USA) with protease inhibitor and phosphatase inhibitor. Each protein concentration of samples was measured using Bradford assay (Protein Assay Dye Reagent Concentrate; Bio Rad, Hercules, CA, USA). The immunostaining was detected using an enhanced chemiluminescence system with PierceTM ECL Western blotting substrate (Thermo Fisher Scientific, Cleveland, OH, USA), following the manufacturer's protocol.

- 1. **Immunohistochemistry**

Deparaffinized tumor specimens were cut into serial consecutive 10 µm slides and the non-cancerous component of the specimen was identified. After deparaffinization, endogenous peroxidase blocking was performed after antigen retrieval with citrate buffer by microwaving. Primary antibodies were rabbit anti-CDK1 (ab265590; Abcam, Cambridge, MA, USA), rabbit anti-KDF1 (PA5-55926; Invitrogen, MA, USA). A detailed protocol is available in supplementary methods. The sections were incubated with primary antibodies at 4℃ for overnight. The slides were incubated with the secondary antibody for 1 hour, washed three times, developed using Peroxidase Substrate DAB kit (SK-4100; Vector Laboratories, Burlingame, CA, USA). Finally, the slides was counterstained with hematoxylin.

1. **Supplementary results**
   1. **Markers representing gastric atrophy and intestinal metaplasia**

Both SOM and Mfuzz classified genes into 16 clusters by OLGA stage. A total of 831 genes were overlapped between SOM and Mfuzz and the expression pattern of 149 genes showed a significant correlation (Spearman correlation coefficient 0.4 and p-values < 5x10-2) with OLGA stage (Supplementary Figure 2D). Out of those, 55 genes showed a significant difference in survival curves based on the Kaplan-Meier method using The Cancer Genome Atlas (TCGA) database (log-rank p-value < 5x10-2) and 30 genes were significantly associated with decreased survival when gene expression was high (Supplementary Table 4). Among them, CAMP-responsive element binding protein 5 (CREB5) was reported to promote cancer invasion and metastasis, and its expression was associated with poor overall survival in The Cancer Genome Atlas gastric cancer dataset (https://portal.gdc.cancer.gov/) (Supplementary Figure 2E).^25,26^

- 1. **Mutational burden is increased in tumor issue of MGC patients**

Somatic variants were investigated from 19 WES samples (Figure 1D). There was one hypermutator (C21) in the control group. The average tumor purity was estimated as 15.4% and 17.2% for control (without the hypermutator) and MGC samples, respectively (Figure 1D). A total of 9,197 somatic variants were identified including 8,788 single-nucleotide variants (SNVs) and 278 small insertions and deletions. The median of nonsynonymous/synonymous mutation (NS/S) ratio was 2.51 and 2.59 and transition/transversion (Ti/Tv) ratio was 3.13 and 2.02 from control and MGC samples (Supplementary Table 5). Mutational signature analysis revealed that the aging signature was predominant in all samples and there are no outstanding differences in signatures between control and MGC (Supplementary Figure 10).

1. **Supplementary Figures**
   1. **Supplementary Figure 1.** Results of bulk RNA sequencing. A. Distribution of gene expression values of the RNA-sequencing data across the samples. B. Principal component analysis (PCA) plot of 23 control samples and 23 MGC samples. C. Sample-to-sample distances were calculated by individuals’ gene expression profiles and samples were hierarchically clustered. D. MA plot shows the dysregulated genes (red) in control and MGC groups.

**
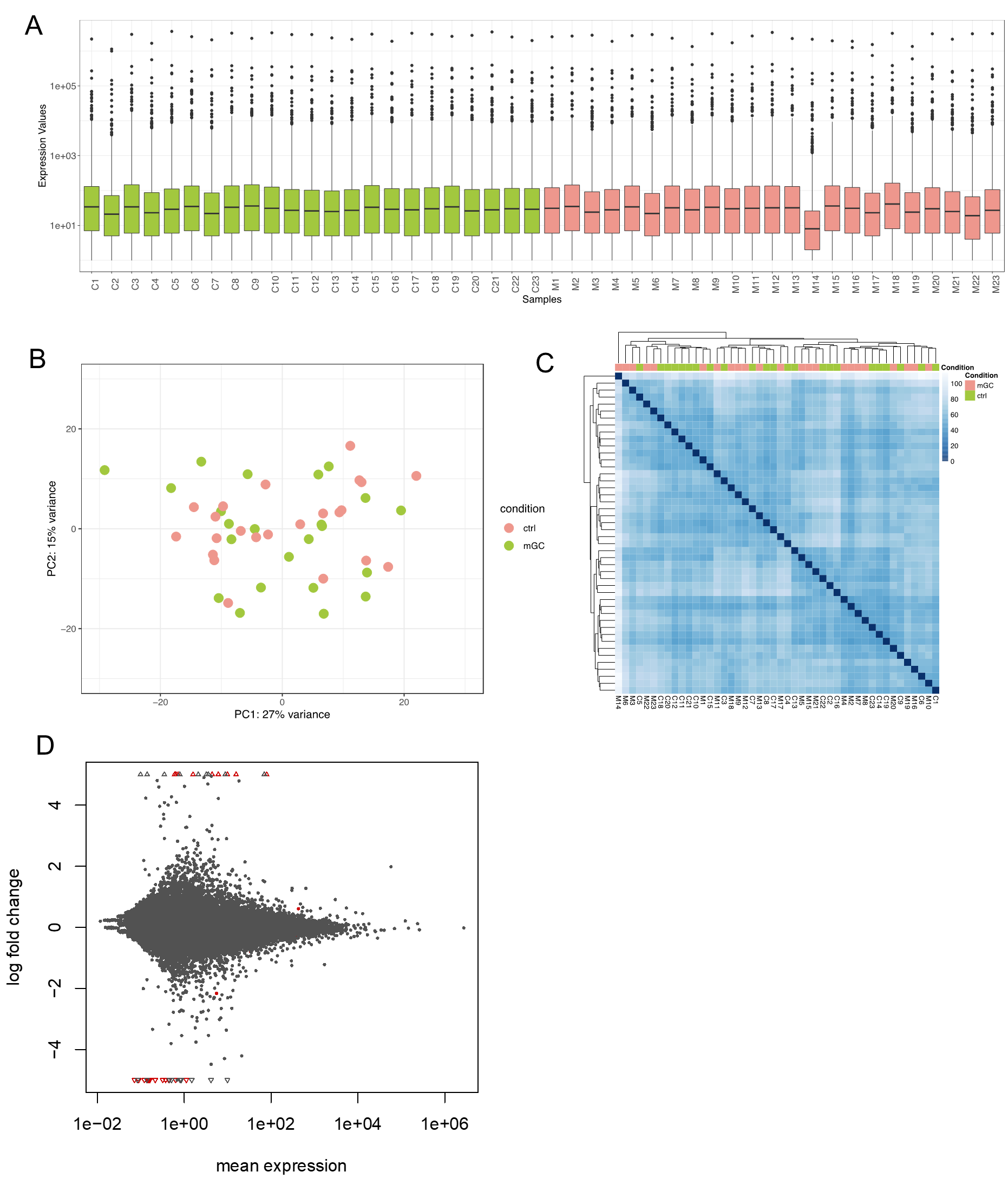
**

- 1. **Supplementary Figure 2**. Gene expression of 13 DEGs and results of SOM/Mfuzz analysis. (A) Gene expression of 13 DEGs in control and MGC groups. (B, C) Clusters defined by the SOM (B) and Mfuzz (C) analysis. Clusters showing the increasing pattern are marked with red circles and boxes. (D) Venn diagram of Number of genes from SOM cluster 3 and 9, Mfuzz cluster 6 and 13. Numbers in bracket indicate the number of genes showing significant correlation (Spearman correlation ≥ 0.4 and p-values < 0.05) with OLGA stage. (D) Kaplan-Meier survival curves according to CREB5 expression in the TCGA gastric cancer dataset (https://portal.gdc.cancer.gov/).


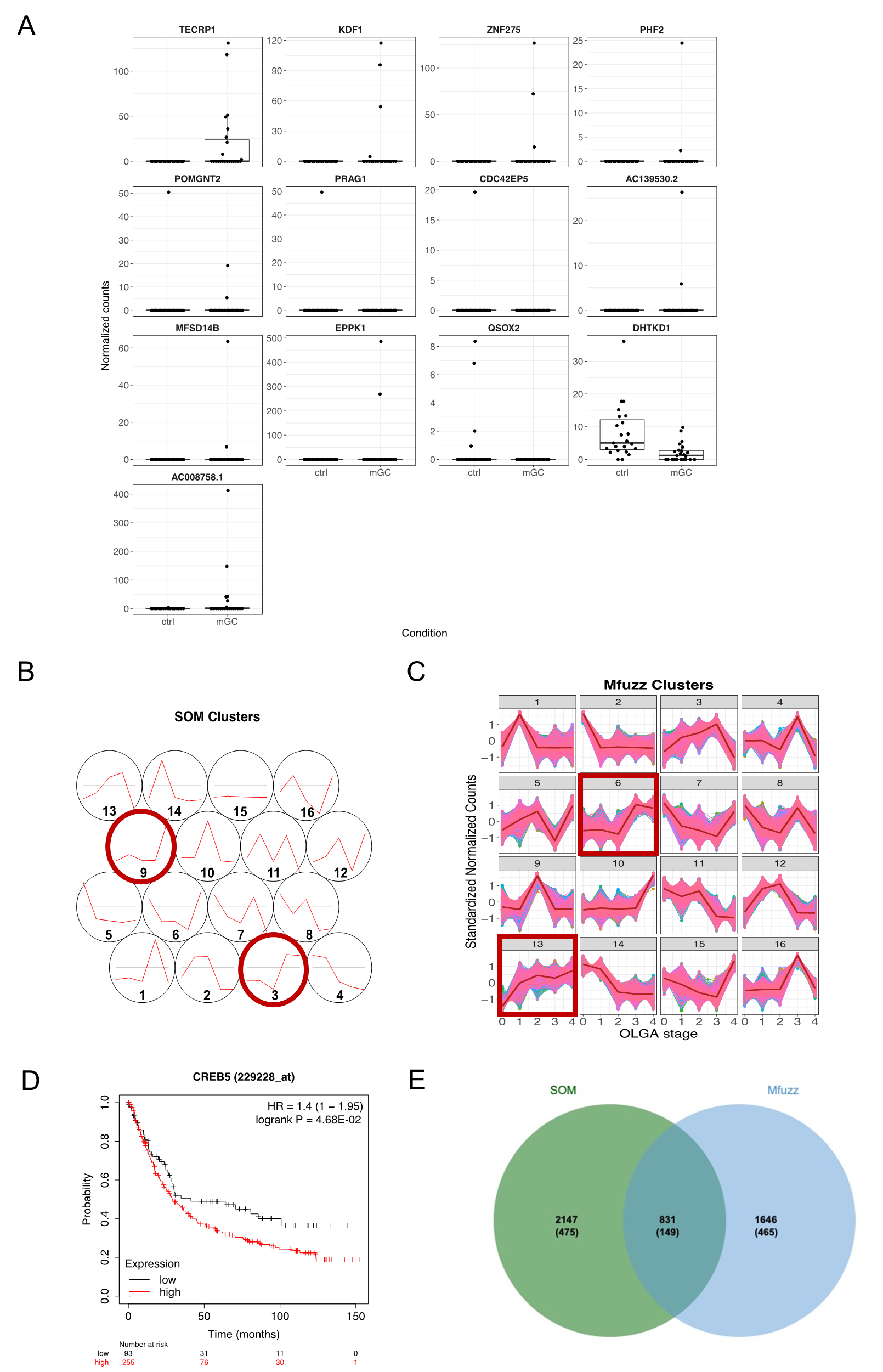


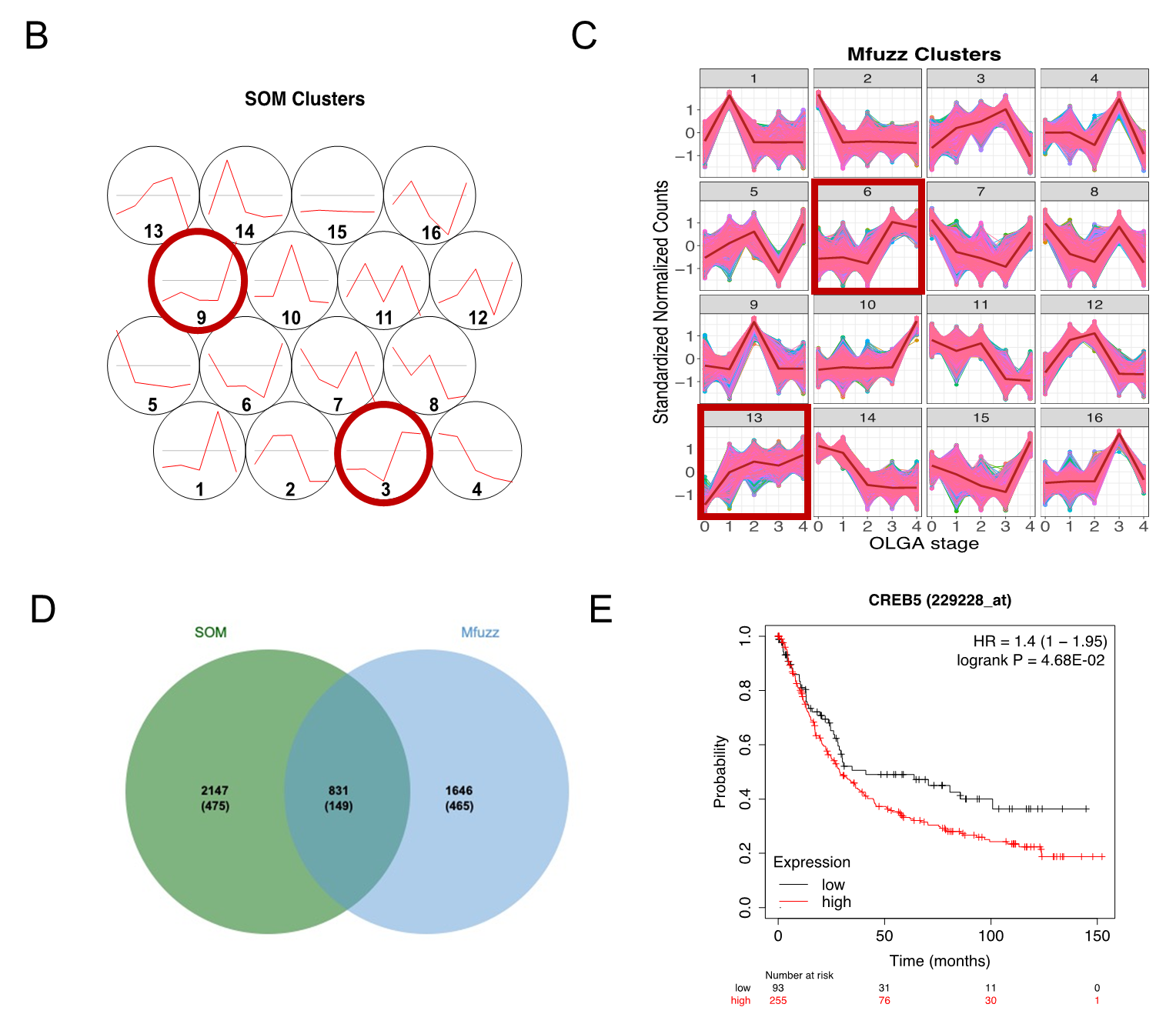


- 1. **Supplementary Figure 3.** Relative expression of candidate genes in various gastric cancer cell lines and normal gastric epithelial cell lines. Increased gene expression of candidate markers in gastric cancer cell lines compared to normal gastric epithelial cell line is noticed.

**
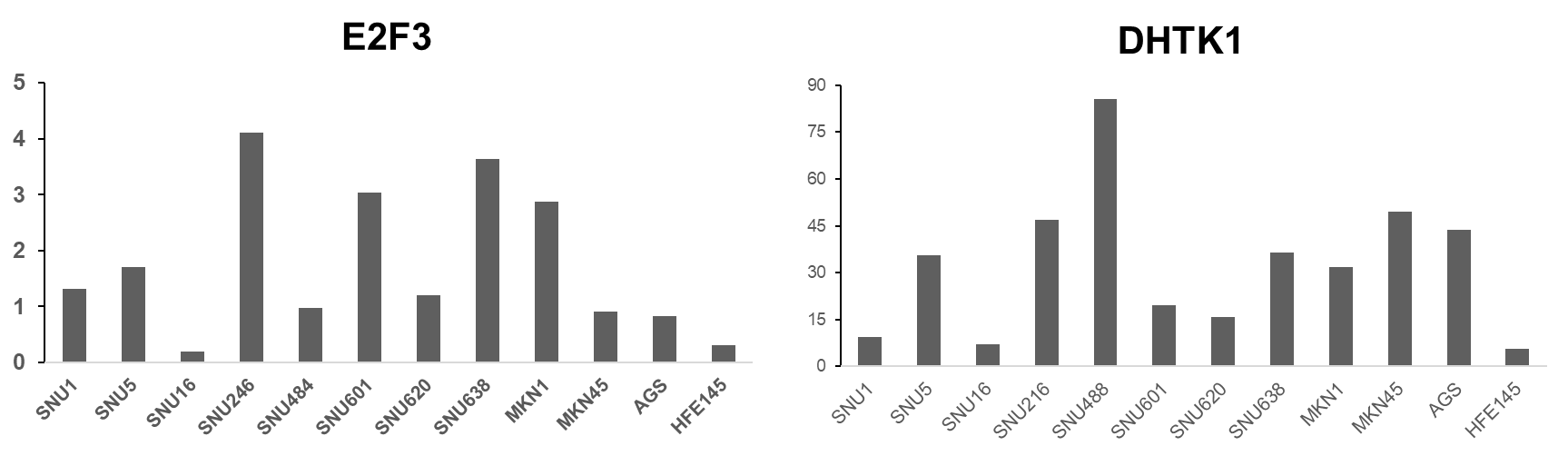
**

- 1. **Supplementary Figure 4.** Knockdown of candidate genes with siRNA transfection (0, 25, and 50 μmol).

**
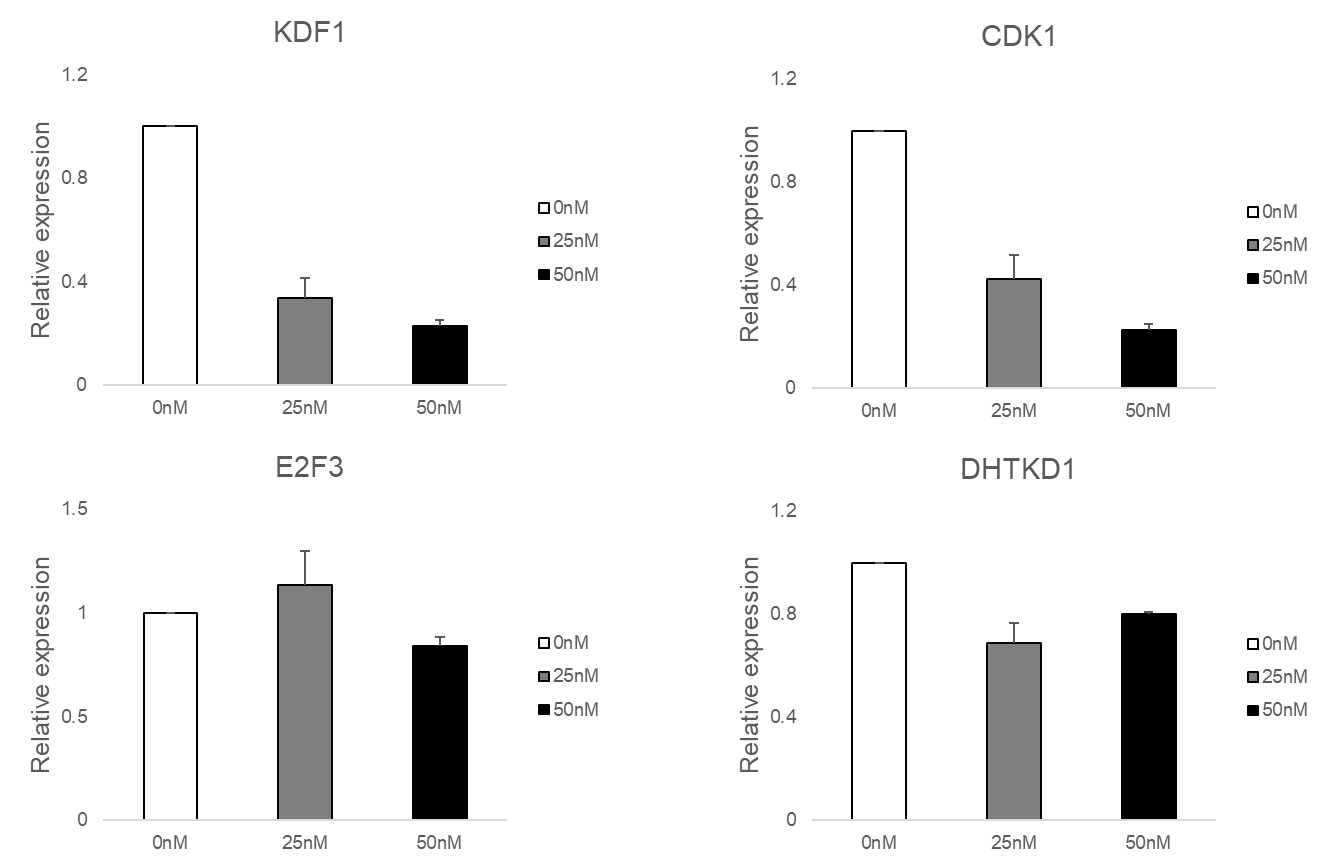
**

- 1. **Supplementary Figure 5**. Expression of KDF1 and CDK1 according to OLGIM stage. (A) Expression 5 of KDF1 according to each OLGIM stage (B) Expression of CDK1 according to each OLGIM stage (C) Expression of KDF1 according to each OLGIM stage classified as low (0-II) and high (III-IV) (D) Expression of CDK1 according to each OLGIM stage classified as low (0-II) and high (III-IV) (E) Expression of KDF1 of MGC patients and controls with subgroup analysis according to low OLGIM stage (0-II) patients and high OLGIM stage (III-IV) patients (F) Expression of CDK1 of MGC patients and controls with subgroup analysis according to low OLGIM stage (0-II) patients and high OLGIM stage (III-IV) patients ***p < 0.0001

**
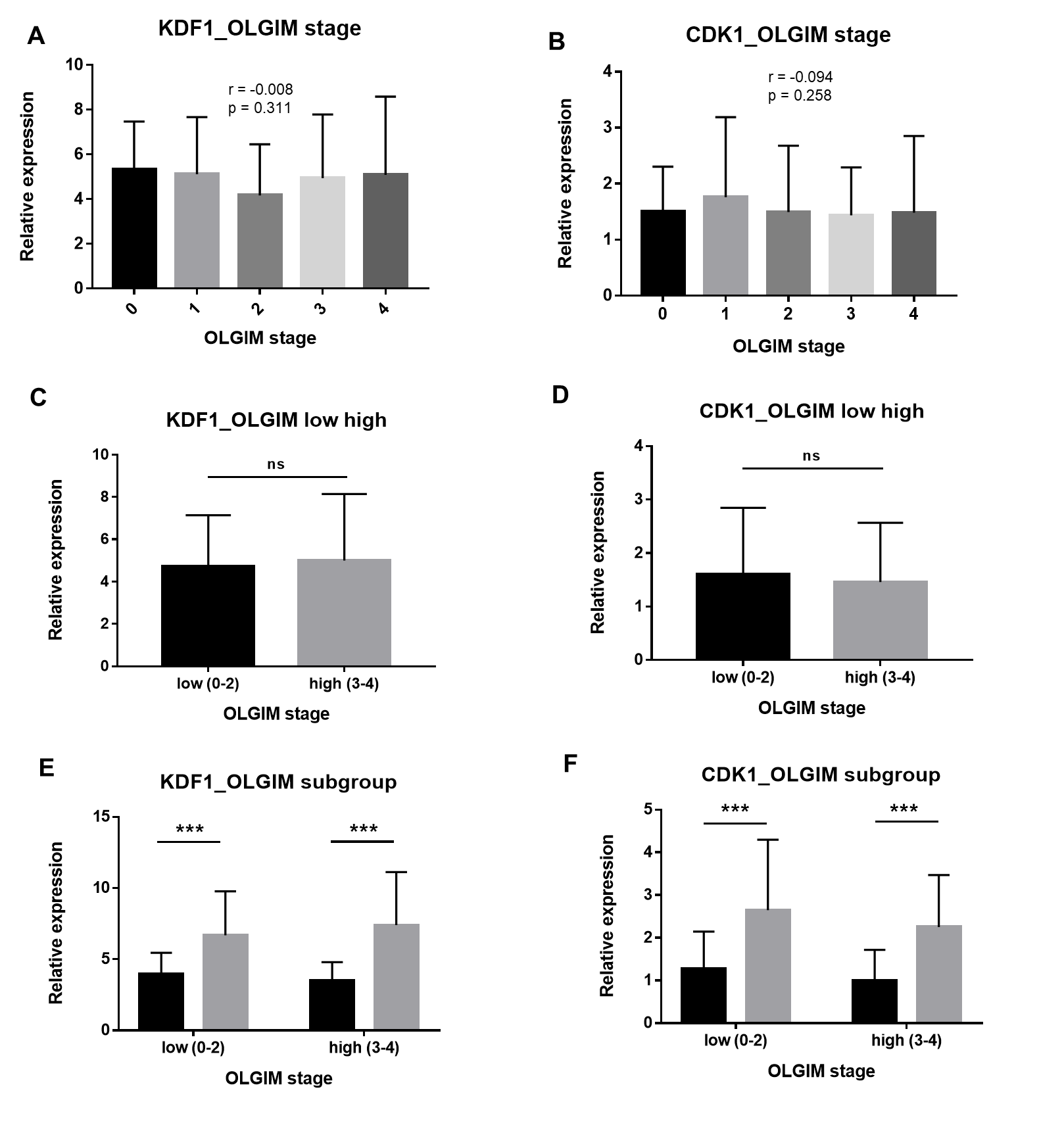
**

- 1. **Supplementary Figure 6.** Expression of CREB5, AKT2 isoform (ENST00000358335.9) and canonical isoform AKT2 according to OLGIM stage. (A-C) Expression levels of (A) CREB5, (B) AKT2 isoform (ENST00000358335.9) and (C) canonical isoform AKT2 measured by RT-qPCR in fresh-frozen normal gastric epithelial tissue of the validation set, and stratified by OLGIM stage 0 to IV. CREB5 and AKT2 shows good correlation with OLGIM stage while canonical isoform AKT2 does not show such significance.

**
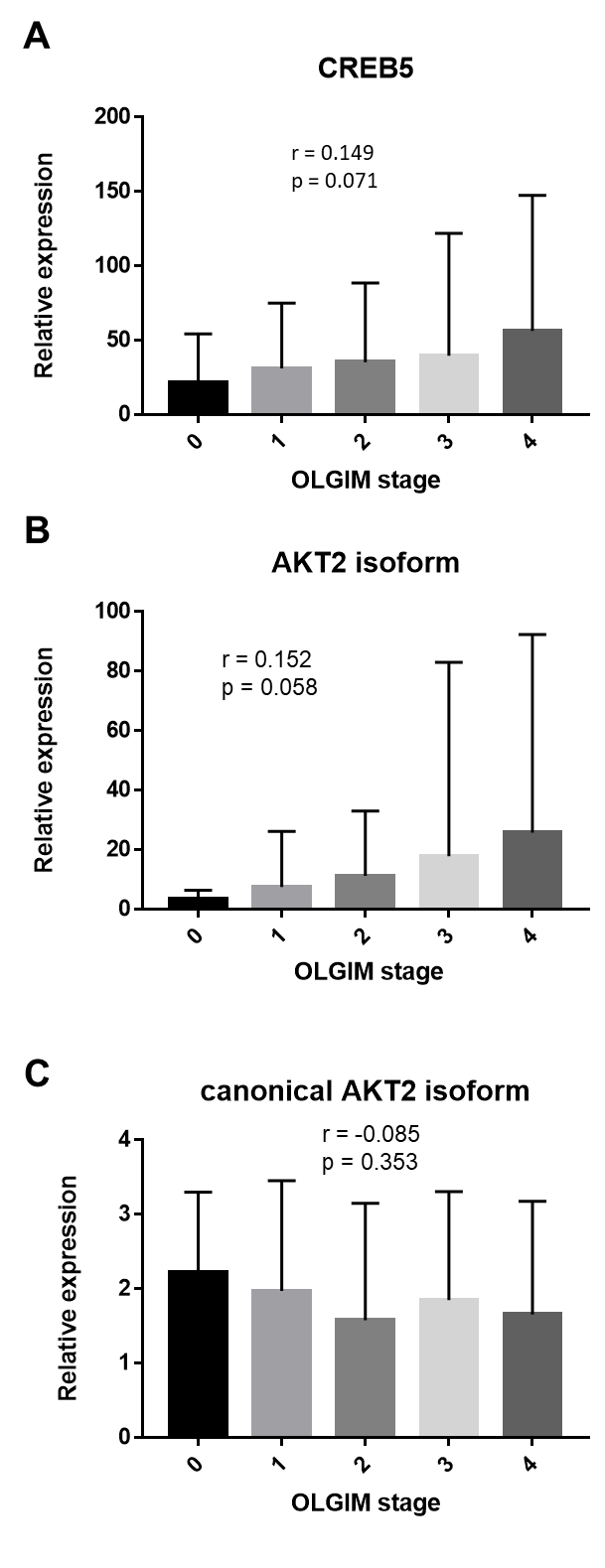
**

- 1. **Supplementary Figure 7**. Module scores consisted of KDF1, CDK1, and CREB5 for all samples and scores are significantly different between control and mGC groups by the Wilcoxon test. Each dots indicate the patients. Supplementary Figure 10. Somatic variants, mutational signatures and frequently detected cancer genes.

- 1. **Supplementary Figure 8.** Somatic variants, mutational signatures and frequently detected cancer genes. (A) Comparison of somatic variants between control and MGC groups and MSI and MSS groups. Comparisons were conducted by the variants classification or type. Hyper-mutator C21 was excluded from the comparison. (B) Mutational signatures of all samples. Samples were hierarchically clustered by the weight of each signature. (C) Frequencies and types of somatic alterations occurred in frequently detected 23 cancer genes.

**
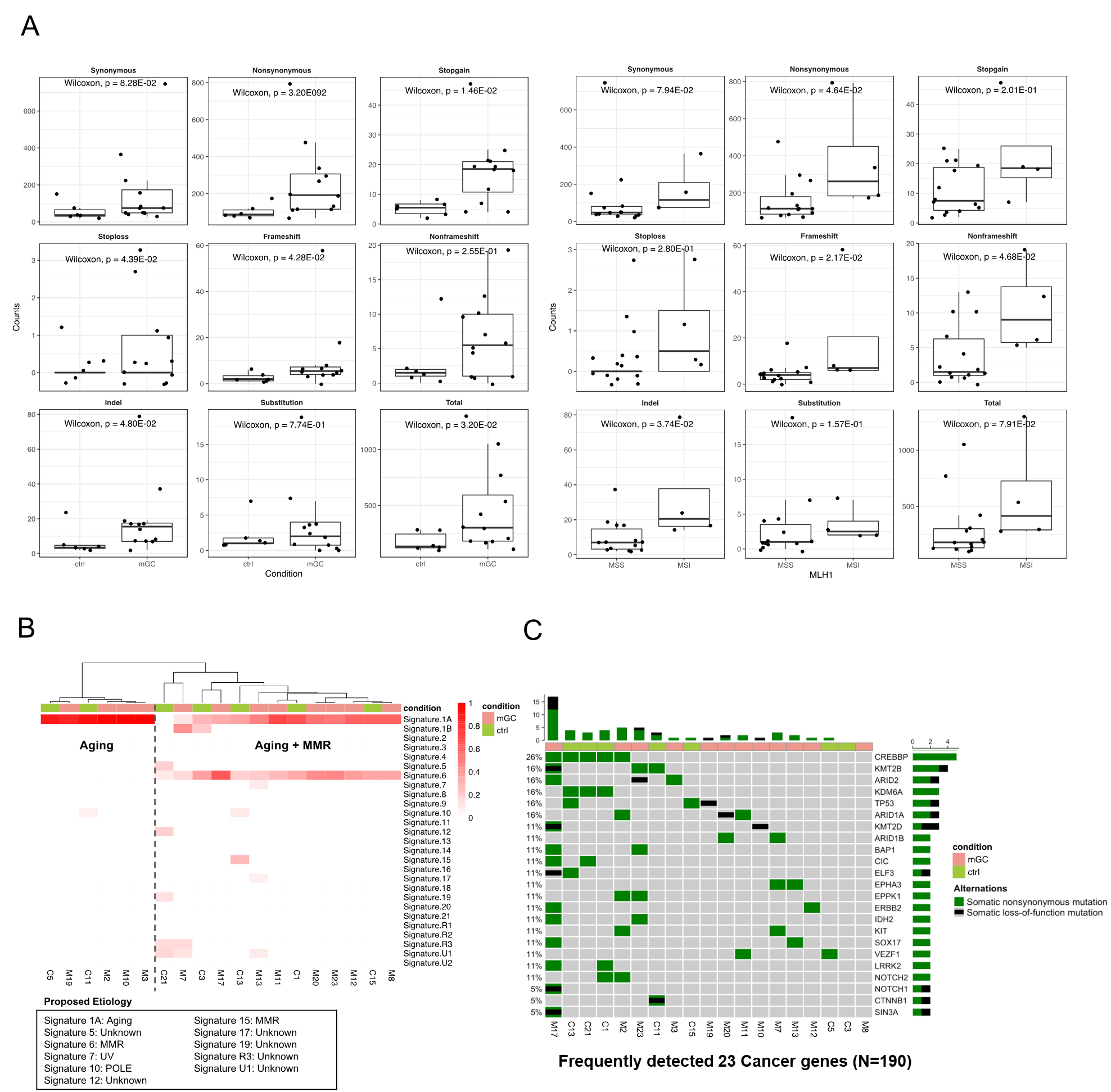
**

1. **Supplementary Tables**
   1. **Supplementary Table 1. Detail of primer sequence for Q-PCR**

| **Gene** | **Forward primer sequence (5’-3’)** | **Reverse primer sequence (5’-3’)** |
| --- | --- | --- |
| KDF1 | GTTCAGCGTACACCAGATCGA | CACGGCTCTTTCGGGTACTAA |
| EPPK1 | ACCTGCTGTCCTCTCCATACT | CCTTGGTTTTGCGTCTCTGTT |
| TNFAIP8L3 (TIPE3) | ACAACAAGAAGGAAGCCCACA | CAGGAGATTGGAGAGCACGTT |
| EMX2 | AGCCCCGAGAGTTTCCTTTTG | TACCTGAGTTTCCGTGAGGCT |
| QSOX2 | AGGGGTGAGGAAGAGGAGAAA | GTACAGCACGACACAGAGACT |
| DHTKD1 | CCTTCCACACGGCAGGATTAT | GGTAAACGTCTCCTTTTGCAGT |
| EGR4 | AGAGTTGTGTGCGGAGCTTT | CTTGCTGTGCCGTTTCTTCTC |
| ZNF488 | GAGCACGAAGTGAGCAAAGAAG | CCAACAAGCGAGGTCTGTAGT |
| DRD4 | GCCTCCATCTTCAACCTGTG | AGCACACGGACGAGTAGACC |
| PCDHB15 | GACACTGGGACAAATGGAGAGA | CCAGCACCTTAACAGAGACAGA |
| AZU1 | ATGCCCGCTTCGTGATGAC | CTGATGGAAAACGTCTGGCG |
| MIB1 (MIB-1) | CCAACAAACTGCCCTACACCT | CATCCACCTTCCCCACATCTT |
| ARIH2 | TGTATGCAGTTTGTGCGAAAGG | GCCATGCAAGAGACTCCCA |
| RFPL3 | GATGCGCTGTCTGCTTCAAG | GTGATGCACCCACTTCGGA |
| SCUBE2 | TGTGGCTCTTCCTCTCCTCTC | AGCTGTGCTTCTCTGGTAGTG |
| ZNF662 | CAAACCTGATTCGTCACCAGA | TGTTGCGTAAGGCTTGTGTTC |
| MAP1B | GACAACACGAACCCCTGATACT | CACAAGAGGACACGAGGCATAA |
| CENPJ | GGAGCCACTTGAACCACTGAA | CCCATCTGCACTCACTTCCTT |
| E2F3 | ATGGACAAAAGGGCACTGCT | CTTTGGAGGGAGGAGGAACAG |
| TMED2 | CGGTCTCGGGCTATTTCGTTA | TCCATCCATGTGAGCAGCAAA |
| TM9SF4 | AGCTGTGTGCAGAGGATTACC | GCCAGAAGGACAAGACCATGA |
| COL3A1 | TTGCTGGGATTGGAGGTGAAA | AAAACCCCGCTAGAAACTGCA |
| CCDC116 | GAAAACGCAAGGACAGAGGAG | TAAAGGCGGTTGGAGAAGTGA |
| TAL1 | TTCACCACCAACAATCGAGTG | GCGGAGGATCTCATTCTTGCT |
| CDK1 | AAACTACAGGTCAAGTGGTAGCC | TCCTGCATAAGCACATCCTGA |
| VPS13B | CTTGGGGAAGAGTGTTGGTCT | TGCTCTGGCTTCTTTCCTTCT |
| CREB5 | CCCTGCCCAACCCTACAATG | GGACCTTGCATCCCCATGAT |
| AKT2 | GGTCGCCAACAGCCTCAA | CACTTTAGCCCGTGCCTTG |
| AKT2-isoform | ﻿CTCCACAAGCGTGGTGAATA | ﻿CAGGCAGAGGGAACAGACAG |

- 1. **Supplementary Table 2. Detail of sequence for siRNA**

| **Gene** | **Sense sequences** | **Antisense sequences** |
| --- | --- | --- |
| KDF1 | GACAGAGCUGUUCAGCGUA | UACGCUGAACAGCUCUGUC |
| CDK1 | CCUGGUCAGUACAUGGAUU | AAUCCAUGUACUGACCAGG |
| E2F3 | GAGCUUGGAGCGAGUCAGU | ACUGACUCGCUCCAAGCUC |
| DHTKD1 | CUGCUUUCCAGUCACUGUA | UACAGUGACUGGAAAGCAG |

- 1. **Supplementary Table 3. Recurrent somatic mutations from MGC (red) and control (blue) patients.**

| **SAMPLE** | **CHR** | **POSITION** | **REF** | **ALT** | **GENE** | **TYPE** | **AA CHANGE** | **dbSNP** | **ExAC** | **gnomAD** | **MetaSVM** | **CADD** | **AD** |
| --- | --- | --- | --- | --- | --- | --- | --- | --- | --- | --- | --- | --- | --- |
| M17 | 14 | 79117583 | G | A | NRXN3 | Mis | p.D383N | . | . | . | T | 35 | 91,4 |
| M2 |  |  |  |  |  |  |  |  |  |  |  |  | 65,4 |
| M17 | 1 | 227261695 | G | A | CDC42BPA | Mis | p.R869C | . | . | . | T | 20.6 | 142,21 |
| M3 |  |  |  |  |  |  |  |  |  |  |  |  | 88,5 |
| M17 | 11 | 55322161 | T | C | OR4C15 | Mis | p.S73P | . | . | . | T | 14.76 | 58,15 |
| M3 |  |  |  |  |  |  |  |  |  |  |  |  | 49,13 |
| M7 | 17 | 56060747 | T | A | **VEZF1** | Mis | p.E14V | . | . | . | T | 13.95 | 105,4 |
| M11 |  |  |  |  |  |  |  |  |  |  |  |  | 204,9 |
| M7 | 17 | 56060749 | A | T |  | Mis | p.H13Q | . | . | . | T | 11.43 | 105,4 |
| M11 |  |  |  |  |  |  |  |  |  |  |  |  | 204,9 |
| M7 | 10 | 27436455 | C | T | YME1L1 | Mis | p.R104H | . | 3.86E-05 | 3.75E-05 | T | 5.39 | 19,4 |
| M12 |  |  |  |  |  |  |  |  |  |  |  |  | 49,6 |
| C21 | 1 | 155891390 | C | T | KHDC4 | Mis | p.V348I | . | . | . | T | 18.68 | 53,8 |
| M7 |  |  |  |  |  |  |  |  |  |  |  |  | 20,4 |
| C21 | 15 | 43028158 | C | T | CDAN1 | Mis | p.A230T | . | . | . | T | 16.45 | 211,46 |
| M7 |  |  |  |  |  |  |  |  |  |  |  |  | 195,17 |
| C21 | X | 54265387 | T | C | WNK3 | Mis | p.K1266R | . | . | . | T | 13.26 | 69,5 |
| M7 |  |  |  |  |  |  |  |  |  |  |  |  | 37,4 |
| C21 | 2 | 8888061 | G | A | KIDINS220 | Mis | p.P1162S | . | . | . | T | 13.11 | 144,24 |
| C5 |  |  |  |  |  |  |  |  |  |  |  |  | 67,5 |
| C15 | 1 | 47610117 | C | T | CYP4A22 | Mis | p.H242Y | rs12754799 | 8.24E-06 | 7.23E-06 | T | 11.57 | 215,10 |
| C13 |  |  |  |  |  |  |  |  |  |  |  |  | 243,9 |
| C21 | 19 | 10795133 | T | G | ILF3 | Mis | p.S701A | . | . | . | T | 11.03 | 171,36 |
| M7 |  |  |  |  |  |  |  |  |  |  |  |  | 60,4 |
| C21 | 15 | 88522659 | G | A | NTRK3 | Mis | p.P586S | . | . | . | T | 10.3 | 53,14 |
| M7 |  |  |  |  |  |  |  |  |  |  |  |  | 29,5 |
| C15 | 15 | 45409813 | T | C | DUOXA1 | Mis | p.D451G | . | . | 0.00E+00 | T | 8.49 | 36,4 |
| C1 |  |  | TC | CT |  |  | p.D451S |  |  | . | . | . | 53,4 |
| C3 | 12 | 99139616 | T | A | ANKS1B | Mis | p.I485F | . | . | 0.00E+00 | T | 8.42 | 165,9 |
| M7 |  |  |  |  |  |  |  |  |  |  |  |  | 105,21 |
| C21 | 8 | 77617362 | C | A | ZFHX4 | Mis | p.H347N | . | . | . | T | 8.23 | 136,32 |
| M7 |  |  |  |  |  |  |  |  |  |  |  |  | 48,6 |
| C21 | 6 | 107475971 | C | G | PDSS2 | Mis | p.R351T | . | . | . | T | 7.53 | 47,4 |
| M7 |  |  |  |  |  |  |  |  |  |  |  |  | 79,5 |
| C21 | 11 | 4107755 | A | T | STIM1 | Mis | p.H508L | . | . | . | T | 6.3 | 411,75 |
| M7 |  |  |  |  |  |  |  |  |  |  |  |  | 24,4 |
| M10 | 19 | 9070789 | T | A | MUC16 | Mis | p.T5553S | . | . | . | T | 5.15 | 14,3 |
| C13 |  |  |  |  |  |  |  |  |  |  |  |  | 64,6 |
| M23 | 17 | 48151888 | G | A | ITGA3 | Mis | p.A487T | . | 8.28E-06 | 4.09E-06 | T | 4.69 | 44,4 |
| C13 |  |  |  |  |  |  |  |  |  |  |  |  | 49,4 |
| C21 | 16 | 84270745 | C | T | KCNG4 | Mis | p.S116N | . | . | . | T | 3.97 | 213,45 |
| M7 |  |  |  |  |  |  |  |  |  |  |  |  | 194,9 |
| C15 | 2 | 97779551 | A | T | ANKRD36 | Mis | p.Q25H | . | 1.00E-04 | 1.16E-04 | T | 1.86 | 218,19 |
| M17 |  |  |  |  |  |  |  |  |  |  |  |  | 219,36 |
| M13 |  |  |  |  |  |  |  |  |  |  |  |  | 80,11 |
| C21 | 11 | 82878581 | C | A | PCF11 | Mis | p.T709K | . | . | . | T | 1.25 | 41,11 |
| M7 |  |  |  |  |  |  |  |  |  |  |  |  | 15,3 |
| C21 | 2 | 200188627 | T | C | SATB2 | Mis | p.I481V | . | . | . | T | 0.8 | 121,32 |
| M7 |  |  |  |  |  |  |  |  |  |  |  |  | 71,9 |
| C21 | 14 | 75266074 | G | C | YLPM1 | Mis | p.E1358D | . | . | . | T | 0.02 | 83,21 |
| M7 |  |  |  |  |  |  |  |  |  |  |  |  | 19,4 |
| C15 | 2 | 97779555 | CC | TA | ANKRD36 | Mis | p.P27Y | . | . | . | . | . | 210,17 |
| M17 |  |  |  |  |  |  |  |  |  |  |  |  | 202,25 |
| M13 |  |  |  |  |  |  |  |  |  |  |  |  | 76,9 |
| C21 | 10 | 76719778 | A | G | KAT6B | Mis | p.N45S | . | . | . | . | . | 80,6 |
| M7 |  |  |  |  |  |  |  |  |  |  |  |  | 76,4 |
| C21 | 1 | 111215877 | TT | GG | KCNA3 | Mis | p.K517K | . | . | . | . | . | 161,22 |
| C3 |  |  |  |  |  |  |  |  |  |  |  |  | 68,3 |

- 1. **Supplementary Table 4. Survival analysis based on the Kaplan-Meier method using The Cancer Genome Atlas (TCGA) of SOM and Mfuzz results. Among 55 genes which showed significant survival difference, 30 genes were significantly associated with decreased survival when gene expression was high.**

| **Gene** | **Log rank *p*-value** | **Direction (shorter live)** |
| --- | --- | --- |
| ZFP36 | 1.60E^-07^ | Low |
| MAN2A1 | 6.40E^-07^ | High |
| CENPF | 1.40E^-06^ | High |
| CEMIP | 9.30E^-06^ | High |
| IGHA2 | 1.20E-^05^ | Low |
| C7 | 2.60E-^05^ | Low |
| EREG | 5.00E^-05^ | High |
| CD302 | 7.40E-^05^ | Low |
| NDUFA9 | 9.20E^-05^ | High |
| RPL37A | 1.40E^-04^ | High |
| KDM1A | 2.10E^-04^ | High |
| H2BC5 | 2.30E^-04^ | High |
| HSPE1 | 2.50E^-04^ | High |
| IMMT | 3.10E^-04^ | High |
| H2BC7 | 4.40E^-04^ | High |
| SLC30A9 | 4.80E^-04^ | Low |
| SERPINB2 | 5.50E^-04^ | High |
| HUS1 | 6.60E^-04^ | High |
| ATP5MG | 7.70E^-04^ | Low |
| NPM1 | 8.40E^-04^ | High |
| BST1 | 1.60E^-03^ | Low |
| KLHL8 | 2.40E^-03^ | Low |
| C12orf42 | 2.70E^-03^ | High |
| DSCAML1 | 3.60E^-03^ | Low |
| EPSTI1 | 3.70E^-03^ | Low |
| G6PC1 | 3.90E^-03^ | High |
| RPL17 | 4.90E^-03^ | Low |
| HOXA7 | 5.50E^-03^ | High |
| DECR1 | 8.00E^-03^ | Low |
| HSFX1 | 1.30E^-02^ | Low |
| LRRC8D | 1.50E^-02^ | High |
| ANO10 | 1.60E^-02^ | Low |
| DERL1 | 1.60E^-02^ | High |
| FAM104B | 1.60E^-02^ | Low |
| DYNC2LI1 | 1.90E^-02^ | Low |
| PSMA2 | 1.90E^-02^ | Low |
| HOXA13 | 2.00E^-02^ | High |
| MRPL34 | 2.00E^-02^ | Low |
| HNRNPC | 2.10E^-02^ | High |
| SRD5A1 | 2.20E^-02^ | High |
| PCK2 | 2.40E^-02^ | Low |
| NUDT1 | 2.50E^-02^ | High |
| EFNA4 | 2.60E^-02^ | High |
| SH3GLB1 | 2.60E^-02^ | Low |
| ATP5MF | 2.70E^-02^ | High |
| RPL27A | 3.10E^-02^ | Low |
| GGT1 | 3.20E^-02^ | Low |
| EXT2 | 3.80E^-02^ | High |
| EEF1B2 | 3.90E^-02^ | Low |
| MIB1 | 3.90E^-02^ | High |
| PAPSS2 | 4.00E^-02^ | Low |
| CREB5 | 4.70E^-02^ | High |
| TC2N | 4.80E^-02^ | High |
| ATP5F1A | 4.90E^-02^ | Low |
| PTCHD1 | 5.00E^-02^ | High |

- 1. **Supplementary Table 5. Variants stats. Stats of total somatic mutations, Non-synonymous/Synonymous mutation ratio (NS/S ratio), transition/transversion ratio (Ti/Tv ratio), and tumor purity of MGC and control patients.**

| **Variable** | | **Control** | **MGC** |
| --- | --- | --- | --- |
| Total somatic mutations | median | 140 | 300 |
|  |  | (4.67/Mb) | (10.00/Mb) |
|  | mean | 526.43 | 459.33 |
|  |  | (17.55/Mb) | (15.31/Mb) |
| NS/S ratio | median | 2.36 | 2.51 |
|  | mean | 2.15 | 2.51 |
| Ti/Tv ratio | median | 1.91 | 3.13 |
|  | mean | 2.08 | 2.98 |
| Purity | median | 0.16 | 0.15 |
|  | mean | 0.16 | 0.17 |

**5. References of supplementary data**

1. Harrow J, Frankish A, Gonzalez JM, et al. GENCODE: the reference human genome annotation for The ENCODE Project. *Genome Res*. Sep 2012;22(9):1760-74. doi:10.1101/gr.135350.111

2. Patro R, Duggal G, Love MI, Irizarry RA, Kingsford C. Salmon provides fast and bias-aware quantification of transcript expression. *Nat Methods*. Apr 2017;14(4):417-419. doi:10.1038/nmeth.4197

3. Soneson C, Love MI, Robinson MD. Differential analyses for RNA-seq: transcript-level estimates improve gene-level inferences. *F1000Res*. 2015;4:1521. doi:10.12688/f1000research.7563.2

4. Love MI, Huber W, Anders S. Moderated estimation of fold change and dispersion for RNA-seq data with DESeq2. *Genome Biol*. 2014;15(12):550. doi:10.1186/s13059-014-0550-8

5. Zhang Y, Parmigiani G, Johnson WE. ComBat-seq: batch effect adjustment for RNA-seq count data. *NAR Genom Bioinform*. Sep 2020;2(3):lqaa078. doi:10.1093/nargab/lqaa078

6. Leek JT, Johnson WE, Parker HS, Jaffe AE, Storey JD. The sva package for removing batch effects and other unwanted variation in high-throughput experiments. *Bioinformatics*. Mar 15 2012;28(6):882-3. doi:10.1093/bioinformatics/bts034

7. Benjamini Y, Hochberg Y. Controlling the False Discovery Rate - a Practical and Powerful Approach to Multiple Testing. *J R Stat Soc B*. 1995;57(1):289-300. doi:DOI 10.1111/j.2517-6161.1995.tb02031.x

8. *EnhancedVolcano: publication-ready volcano plots with enhanced colouring and labeling*. Version R version 1.8.0 2020.

9. Murtagh F, Legendre P. Ward's Hierarchical Agglomerative Clustering Method: Which Algorithms Implement Ward's Criterion? *J Classif*. Oct 2014;31(3):274-295. doi:10.1007/s00357-014-9161-z

10. *Package ‘pheatmap’*. Version 1.0.12 CRAN; 2018.

11. Wehrens R, Buydens LMC. Self- and super-organizing maps in R: The kohonen package. *J Stat Softw*. Oct 2007;21(5):1-19.

12. Hathaway RJ, Bezdek JC. Local Convergence of the Fuzzy C-Means Algorithms. *Pattern Recogn*. 1986;19(6):477-480. doi:Doi 10.1016/0031-3203(86)90047-6

13. Gyorffy B, Surowiak P, Budczies J, Lanczky A. Online Survival Analysis Software to Assess the Prognostic Value of Biomarkers Using Transcriptomic Data in Non-Small-Cell Lung Cancer. *Plos One*. Dec 18 2013;8(12)doi:ARTN e82241

10.1371/journal.pone.0082241

14. Szasz AM, Lanczky A, Nagy A, et al. Cross-validation of survival associated biomarkers in gastric cancer using transcriptomic data of 1,065 patients. *Oncotarget*. Aug 2 2016;7(31):49322-49333. doi:10.18632/oncotarget.10337

15. Vitting-Seerup K, Sandelin A. IsoformSwitchAnalyzeR: analysis of changes in genome-wide patterns of alternative splicing and its functional consequences. *Bioinformatics*. Nov 1 2019;35(21):4469-4471. doi:10.1093/bioinformatics/btz247

16. Hao YH, Hao S, Andersen-Nissen E, et al. Integrated analysis of multimodal single-cell data. *Cell*. Jun 24 2021;184(13):3573-+. doi:10.1016/j.cell.2021.04.048

17. McKenna A, Hanna M, Banks E, et al. The Genome Analysis Toolkit: A MapReduce framework for analyzing next-generation DNA sequencing data. *Genome Research*. Sep 2010;20(9):1297-1303. doi:10.1101/gr.107524.110

18. Van der Auwera GA, Carneiro MO, Hartl C, et al. From FastQ data to high confidence variant calls: the Genome Analysis Toolkit best practices pipeline. *Curr Protoc Bioinformatics*. 2013;43:11 10 1-11 10 33. doi:10.1002/0471250953.bi1110s43

19. Li H. Aligning sequence reads, clone sequences and assembly contigs with BWA-MEM. *arXiv*. 2013:preprint arXiv:1303.3997. doi:https://doi.org/10.48550/arXiv.1303.3997

20. Benjamin D, Sato T, Cibulskis K, Getz G, Stewart C, Lichtenstein L. Calling Somatic SNVs and Indels with Mutect2. *bioRxiv*. 2019:861054. doi:10.1101/861054

21. Wang K, Li M, Hakonarson H. ANNOVAR: functional annotation of genetic variants from high-throughput sequencing data. *Nucleic Acids Res*. Sep 2010;38(16):e164. doi:10.1093/nar/gkq603

22. Rosenthal R, McGranahan N, Herrero J, Taylor BS, Swanton C. deconstructSigs: delineating mutational processes in single tumors distinguishes DNA repair deficiencies and patterns of carcinoma evolution. *Genome Biology*. Feb 22 2016;17doi:ARTN 31

10.1186/s13059-016-0893-4

23. Gu ZG, Eils R, Schlesner M. Complex heatmaps reveal patterns and correlations in multidimensional genomic data. *Bioinformatics*. Sep 15 2016;32(18):2847-2849. doi:10.1093/bioinformatics/btw313

24. Kang S, Park M, Cho JY, et al. Tumorigenic mechanisms of estrogen and Helicobacter pylori cytotoxin-associated gene A in estrogen receptor alpha-positive diffuse-type gastric adenocarcinoma. *Gastric Cancer*. Jul 2022;25(4):678-696. doi:10.1007/s10120-022-01290-0

25. The Cancer Genome Atlas (TCGA) Research Network. https://www.cancer.gov/tcga

26. Wang S, Qiu J, Liu L, et al. CREB5 promotes invasiveness and metastasis in colorectal cancer by directly activating MET. *J Exp Clin Cancer Res*. Aug 25 2020;39(1):168. doi:10.1186/s13046-020-01673-0
